# A new large squalodelphinid (Cetacea, Odontoceti) from Peru sheds light on the Early Miocene platanistoid disparity and ecology

**DOI:** 10.1098/rsos.172302

**Published:** 2018-04-18

**Authors:** Giovanni Bianucci, Giulia Bosio, Elisa Malinverno, Christian de Muizon, Igor M. Villa, Mario Urbina, Olivier Lambert

**Affiliations:** 1 Dipartimento di Scienze della Terra, Università di Pisa, Pisa, Italy; 2 Dipartimento di Scienze dell'Ambiente e del Territorio e di Scienze della Terra, Università di Milano-Bicocca, Milan, Italy; 3 Département Origines et Évolution, CR2P (CNRS, MNHN, UPMC), Muséum National d'Histoire Naturelle, Paris, France; 4 Departamento de Paleontologia de Vertebrados, Museo de Historia Natural de la Universidad Nacional Mayor de San Marcos, Lima, Peru; 5 DO Terre et Histoire de la Vie, Institut Royal des Sciences Naturelles de Belgique, Brussels, Belgium

**Keywords:** Odontoceti, Squalodelphinidae, Early Miocene, Peru, phylogeny, palaeoecology

## Abstract

The South Asian river dolphin (*Platanista gangetica*) is the only extant survivor of the large clade Platanistoidea, having a well-diversified fossil record from the Late Oligocene to the Middle Miocene. Based on a partial skeleton collected from the Chilcatay Formation (Chilcatay Fm; southern coast of Peru), we report here a new squalodelphinid genus and species, *Macrosqualodelphis ukupachai*. A volcanic ash layer, sampled near the fossil, yielded the ^40^Ar/^39^Ar age of 18.78 ± 0.08 Ma (Burdigalian, Early Miocene). The phylogenetic analysis places *Macrosqualodelphis* as the earliest branching squalodelphinid. Combined with several cranial and dental features, the large body size (estimated body length of 3.5 m) of this odontocete suggests that it consumed larger prey than the other members of its family. Together with *Huaridelphis raimondii* and *Notocetus vanbenedeni*, both also found in the Chilcatay Fm, this new squalodelphinid further demonstrates the peculiar local diversity of the family along the southeastern Pacific coast, possibly related to their partition into different dietary niches. At a wider geographical scale, the morphological and ecological diversity of squalodelphinids confirms the major role played by platanistoids during the Early Miocene radiation of crown odontocetes.

## 1. Introduction

Echolocating toothed whales (Cetacea, Odontoceti) experienced an initial significant radiation during the Oligocene, being well documented by a consistent and continuously improving fossil record [1–11]. This broad diversity and morphological disparity was deeply sifted by selection near the Oligocene–Miocene boundary, when most of the stem odontocetes disappeared. Apparently, these odontocete extinctions were selective, because all the extinct clades are characterized by a markedly heterodont dentition (i.e. with double-rooted cheek teeth, often bearing accessory denticles) [12]. During the Early Miocene, this extinction phase was followed by the diversification of several groups of crown odontocetes in the marine environment, generally with an origin during the Late Oligocene. In particular, this first crown odontocete radiation is characterized by a significant diversification of nearly homodont members of the superfamily Platanistoidea (Allodelphinidae, Platanistidae and Squalodelphinidae), forming a monophyletic group in several recent phylogenetic analyses [13–15]. This initial radiation was followed by successive extinction phases during the Middle to Late Miocene, with only the freshwater South Asian dolphin (*Platanista gangetica* Lebeck, 1801 [16]) surviving today [17,18].

With its rich, but only partly described odontocete fauna [13,19–21], the Chilcatay Formation (Chilcatay Fm) (Pisco Basin, southern coast of Peru) represents an important window of the cetacean evolutionary history, providing a unique opportunity to better understand the crucial, early steps of the first radiation of crown Odontoceti. Indeed, based on published and ongoing stratigraphic studies, the fossiliferous beds of this formation prove to have been deposited during a short interval of time, roughly between 19 and 18 Myr (early Burdigalian, Early Miocene) in the Western Ica Valley [20,22].

A significant part of the Chilcatay fossil assemblage was referred to the Squalodelphinidae, an Early Miocene family that can be distinguished from the other members of the Platanistoidea in having a moderately elongated and tapered rostrum, single-rooted posterior teeth retaining ornamentation and marked skull asymmetry.

The previously described squalodelphinid remains from the Chilcatay Fm, all from the Ullujaya locality, were referred to the diminutive new genus and species *Huaridelphis raimondii* Lambert *et al*. 2014 [13] and to the larger *Notocetus vanbenedeni* Moreno, 1892 [23], a species already known from the Early Miocene of Argentina [13,21].

A new partial skeleton of platanistoid was recently collected in a new Chilcatay Fm outcrop located near Cerro Colorado, a locality from the overlying Late Miocene Pisco Formation well known for its rich fossil content [24].

This new specimen from the Chilcatay Fm belongs to a new squalodelphinid, *Macrosqualodelphis ukupachai*, differing from all the other genera of the family in several characters, including size, the more robust rostrum and the larger temporal fossa.

The aim of this paper is to describe this new squalodelphinid and to investigate its phylogenetic relationships, to provide an accurate bio- and chronostratigraphic setting and to analyse the ecological and evolutionary significance of this new record, both from a local and worldwide viewpoint.

## 2. Material and methods

### 2.1. Institutional abbreviations

BDNLTM, Bünde Doberg und Tabak Museum (Bünde, Germany); MGP, Museo di Geologia e Paleontologia dell'Università di Padova; MLP, Museo de Ciencias Naturales de La Plata (Buenos Aires, Argentina); MUSM, Museo de Historia Natural, Universidad Nacional Mayor de San Marco (Lima, Peru); USNM, National Museum of Natural History, Smithsonian Institution (Washington, DC: USA).

### 2.2. Anatomical abbreviations

BZW, bizygomatic width of the skull; CBL, condylobasal length of the skull; TBL, total body length.

### 2.3. Anatomical terminology

The anatomical terminology follows Mead & Fordyce [25] for the skull and mostly Evans & de Lahunta [26] for the postcranial skeleton.

### 2.4. Cladistic analysis

The phylogenetic relationships of *Macrosqualodelphis* with the other nearly homodont platanistoids are investigated here, using the matrix published by Lambert *et al*. [13] and recently modified by Godfrey *et al*. [15], with only a few additions.

Concerning the taxon list, in addition to *Macrosqualodelphis*, we coded the platanistid *Dilophodelphis* from the Early Miocene of Oregon (USA), recently described by Boersma & Pyenson [14] (see appendix A).

As for the character list, we added a new character, whose derived state is shared by *Huaridelphis*, *Notocetus* and *Squalodelphis*:

Left frontal longitudinally shorter than the right frontal at the vertex due to the shift of the vertex on the right side: absent (0); present (1).

After these additions, the matrix includes 23 taxa coded for 42 morphological characters.

The parsimony analysis was executed with the software paup (v. 4.0b10; [27]), considering all characters unordered, and using the tree bisection and reconnection (TBR) algorithm optimized by ACCTRAN.

To further support the referral of *Macrosqualodelphis* to the family Squalodelphinidae, we included this taxon in the matrix of Tanaka & Fordyce [6], recently modified by Lambert *et al*. [20]. We also coded *Huaridelphis*, and some characters of *Notocetus* and *Squalodelphis* were coded differently from previous versions of the matrix (see Lambert *et al.* [20] for methods and appendix B for the coding of the characters of *Huaridelphis*, *Macrosqualodelphis* and *Notocetus*). We performed four parsimony analyses, also using the software paup (v. 4.0b10; [27]), considering all characters unordered and unweighted, and using the TBR algorithm optimized by ACCTRAN: a first analysis with equally weighted characters and without molecular constraint; a second analysis with down-weighted homoplastic characters, following the method of Goloboff [28] with the constant *k* > 3 and without molecular constraint; a third analysis with equally weighted characters and with a backbone molecular constraint taken from the analysis of McGowen *et al*. [29], as performed by Tanaka & Fordyce [6] and a fourth analysis with down-weighted homoplastic characters and with a backbone molecular constraint.

### 2.5. Body size

To estimate the TBL of MUSM 2545 and of all other fossil platanistoids with single-rooted posterior teeth included in our phylogenetic analysis, we used the equation provided by Pyenson & Sponberg [30] for stem Platanistoidea, based on the BZW:

log(TBL)=0.92×(log(BZW)−1.51)+2.49

Based on the estimated TBL and the 50% majority-rule consensus tree obtained in our phylogenetic analysis as a backbone, we investigated changes in body size among squalodelphinids and related platanistoids using mesquite v. 2.74 [31]. For this analysis, the TBL was considered as an ordered character, including three distinct states that we defined by placing the division in correspondence with the largest gaps of our sample.

### 2.6. ^40^Ar/^39^Ar isotopic analysis

To obtain an absolute dating through the ^40^Ar/^39^Ar method, a tephra layer CHILC-AT1 was sampled in the Chilcatay Fm outcropping 1.7 km southeast (SE) to the locality of the *M. ukupachai* holotype. The sample locality (geographical coordinates: 14°23′49.85′′ S, 75°53′27.35′′ W) is 150 m SE to an uncollected skeleton that most likely belongs to the same species as the *M. ukupachai* holotype.

After sieving, biotite crystals larger than 250 µm were separated by hand-picking under a stereoscopic microscope and a sample of 17.87 mg was selected for dating. Electron Probe Micro-Analyses (EPMAs) were performed using a JEOL 8200 Super Probe at the University of Milan to characterize the chemical composition of the tephra glass and to check for the lack of alteration of the biotite phenocrysts. For ^40^Ar/^39^Ar dating, the biotite sample was irradiated in the nuclear reactor at McMaster University (Ontario, Canada); samples of the Fish Canyon sanidine with the known age of 28.172 ± 0.028 Ma [32] were used as standards during irradiation (the calculated value of the *J* factor was 0.000806). Ar isotope analyses were done on the NuInstruments™ Noblesse® noble gas mass spectrometer at the University of Milano-Bicocca. Step-heating followed the protocol described by Villa *et al*. [33].

### 2.7. Biostratigraphical analysis

Ten sediment samples, collected for biostratigraphical purposes along a 3 m thick section measured around the *Macrosqualodelphis* holotype, were barren of microfossils. Biostratigraphical information is thus derived from samples collected in the Chilcatay Fm at other localities of the Western Ica Valley area (Cerro Submarino, Roca Negra and Ullujaya).

Sediment samples were prepared as smear slides and analysed using an Olympus BX50 polarized optical microscope at 1000×.

## 3. Systematic palaeontology

Cetacea Brisson, 1762

Neoceti Fordyce and Muizon, 2001

Odontoceti Flower, 1867

Platanistoidea Gray, 1863

Squalodelphinidae Dal Piaz, 1917

**Type genus**. *Squalodelphis* Dal Piaz, 1917

**Other genera included.** *Huaridelphis*, *Medocinia*, *Notocetus*, *Phocageneus*.

*Macrosqualodelphis*, gen. nov.

**LSID**: zoobank.org:act:D50FCFB4-AF9E-40AC-85CF-42FA4DA78D18

**Type and only known species**. *Macrosqualodelphis ukupachai*, sp. nov.

**Diagnosis**. As for the type species.

**Etymology**. From ‘*Macro*’, large, and ‘*Squalodelphis*’ the type genus of the family. Gender masculine.

*Macrosqualodelphis ukupachai*, sp. nov.

Figures 2–11, tables 1 and 2.

**LSID**: zoobank.org:act:17E82A17–2C4B-4A2F-B6E2-1BC20A066D09

**Holotype and only referred specimen**. MUSM 2545 consists of a skull lacking the anterior portion of the rostrum, the ear bones, both mandibles and the hyoid bones. The ventralmost portion of the rostrum and of the basicranium is worn along a plane slightly anterodorsally sloping with respect to the horizontal plane of the skull (erupted portion of maxillary teeth, basioccipital crests, ventral part of exoccipitals and postglenoid processes of squamosals missing). MUSM 2545 also preserves three detached anterior teeth; the atlas, two thoracic, two lumbar and eight caudal vertebrae; the left humerus, radius and incomplete ulna; one phalanx and one metacarpal; and two small fragments of ribs.

**Figure 1.**
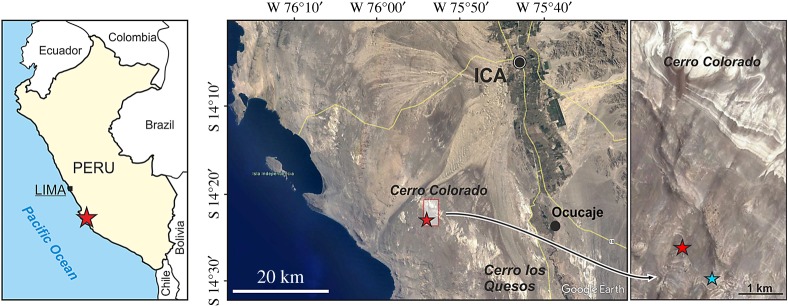
Geographical position (red star) of the locality of the holotype skeleton (MUSM 2545) of *M. ukupachai* in the Western Ica Valley, Ica Region, southern Peru. Blue star indicates the position of the volcanic ash layer sample radiometrically dated (^40^Ar/^39^Ar) to 18.78 ± 0.08 Ma (late Early Miocene, early Burdigalian). This ash layer is close to another uncollected skeleton most likely belonging to the same species.

**Type locality**. About 3 km south of the fossiliferous Cerro Colorado locality [24], Western Ica Valley, Ica Region, southern Peru (figure 1). Geographical coordinates: 14°23′01.9′′ S, 75°53′58.8′′ W; 710 m above sea level. The holotype was discovered and collected by one of the authors (M.U.).

**Type horizon**. The holotype of *M. ukupachai* MUSM 2545 was discovered in the Chilcatay Fm, dated from the latest Oligocene to the earliest Middle Miocene on the basis of diatoms, foraminifers and molluscs [34–37]. In the Western Ica Valley area, the age of this formation can be constricted to the Early Miocene (approx. 19–18 Ma), through biostratigraphic and ^40^Ar/^39^Ar analyses ([20,22], new data). The age of MUSM 2545 is here further constricted to 18.78 ± 0.08 Ma (early Burdigalian) on the basis of a volcanic ash layer sample dated by ^40^Ar/^39^Ar. Although the fragmentation of outcrops (characterized by small isolated patches of Chilcatay Fm) in this area of the Pisco Basin did not allow for a precise correlation of the horizon of the *M. ukupachai* holotype with the dated ash layer, strong similarities between the lithologies outcropping in the two areas and especially the discovery near the ash layer of another (uncollected) skeleton most likely belonging to the same species as MUSM 2545 supports the age of 18.78 ± 0.08 Ma provided for the *M. ukupachai* holotype.

**Diagnosis**. *Macrosqualodelphis* differs from all the other squalodelphinids in: larger size (as seen in BZW and CBL, see below); less abrupt anterior tapering of rostrum in dorsal view (see quantification below); U-shaped left antorbital notch; prominent nuchal crest, higher than the frontals and nasals at the vertex; thinner, blade-like lateral margin of the posterior portion of the rostrum; more voluminous temporal fossa and larger teeth (see quantification below). It further differs from *Huaridelphis*, *Notocetus* and *Squalodelphis* in the right and left frontals displaying roughly the same longitudinal length at the vertex. It further differs from *Huaridelphis* in medial and lateral borders of the right antorbital notch drawing a more open angle (see quantification below); thicker antorbital process of the frontal, distinctly thicker than the antorbital process of the maxilla in lateral view; more elongated postorbital process of the frontal; nasal dorsally inflated; vertex not anteroventrally sloping; vertex more transversely constricted posterior to the nasals (see quantification below); left maxilla descending more abruptly lateroventrally from the vertex, forming a deeper fossa posterolateral to the left nasal; anteroposteriorly longer zygomatic process of the squamosal and probably lower upper tooth count. It further differs from *Notocetus* in smaller nasal and probably lower upper tooth count. It further differs from *Medocinia* and *Squalodelphis* in: dorsal opening of the mesorostral groove being narrower than the premaxilla at the base of the rostrum and wider dorsal exposure of the maxilla at the base of the rostrum (the premaxilla nearly reaches the lateral margin of the rostrum in *Medocinia* and *Squalodelphis*). It further differs from *Squalodelphis* in the transversely wider nuchal crest (crest approximately as wide as the greatest width of the premaxillae in *Squalodelphis* and considerably wider in *Macrosqualodelphis*).

**Etymology**. From ‘*Uku Pacha*’ (*Uku* = within, inside; *Pacha* = Earth), the Inca lower world, located below the Earth's surface, in reference to the discovery of the specimen buried in sediment.

## 4. Description and comparison

### 4.1. Ontogeny

We consider the holotype of *M. ukupachai* as an adult animal, having all epiphyses of preserved vertebrae, humerus, radius, ulna and manus bones strongly fused, and displaying significant apical wear on the crown of the only preserved complete teeth.

### 4.2. Total body length estimate

The TBL of *Macrosqualodelphis* was estimated to 3.50 m, using a BZW value of 370 mm in the Pyenson & Sponberg equation [30].

Using the same equation, we obtained values significantly smaller for the other squalodelphinids: *H. raimondii*, BZW = 207 mm, TBL = 2.05 m; *N. vanbenedeni*, BZW = 254 mm, TBL = 2.47 m and *Squalodelphis fabianii* Dal Piaz, 1917 [38], BZW = 263 mm, TBL = 2.55 m. The estimated TBL of *Macrosqualodelphis* is also much larger than in the extant South Asian river dolphin *P. gangetica*, reaching 2.2 m in adult males and 2.6 m in adult females [39].

### 4.3. Cranium

#### 4.3.1. General morphology

With a CBL greater than 770 mm and a BZW of 370 mm (table 1), the cranium of *M. ukupachai* is larger than in all other known squalodelphinids (*H. raimondii* CBL = 494 mm, BZW = 207 mm; *N. vanbenedeni* CBL = 582–634 mm, BZW = 235–254 mm; *S. fabianii*: CBL = 640 mm, BZW = 263 mm). The skull of the holotype of *Medocinia tetragorhina* Delfortrie, 1875 [40] is too fragmentary to provide estimates of these measurements, but other skull measurements, for example the width at rostrum base, are smaller than in *Macrosqualodelphis*. The original CBL of *Macrosqualodelphis* can be tentatively estimated as a percentage of the CBL within the ranges of the other squalodelphinids (63–70%) [13]. Using these percentages, we obtain an estimated rostrum length for *Macrosqualodelphis* varying between 488 and 644 mm, with the lower value smaller than the preserved rostrum length (490 mm). Using the higher value, the estimated missing anterior portion is 154 mm and the estimated CBL is 924 mm.

**Table 1.** Measurements of the cranium of *M. ukupachai* holotype (MUSM 2545) compared with two other crania of squalodelphinids from the Chilcatay Fm (Early Miocene, Peru) referred to *N. vanbenedeni* and *H. raimondii* (holotype). All measurements are in mm.

	*Macrosqualodelphis ukupachai* MUSM 2545	*Notocetus vanbenedeni* MUSM 1395	*Huaridelphis raimondii* MUSM 1396
condylobasal length	+770	600	494
length of rostrum	+490	403	330
length of neurocranium	280	197	165
width of rostrum at base of rostrum	170	136	92
width of premaxillae at base of rostrum	112	78	55
orbital width of skull	285	227	173
postorbital width of skull	325	—	183
bizygomatic width of skull	370	—	207
maximum width between temporal crests	190	145	128
minimum posterior distance temporal crests	148	134	109
length of orbit	90	55	55
height of temporal fossa	150	66	59
length of temporal fossa	210	108	81
squamosal length	e137	91	—
maximum width premaxillae on neurocranium	141	e105	73
width left premaxillary sac fossa	39	41	27
width right premaxillary sac fossa	41	40	28
maximum distance between premaxillae anterior to nares	20.5	e15	9
width bony nares	57	45	33
anterior width of nasals	51	43	28
length of medial suture of nasals	21	14	10
length of medial suture of frontals at vertex	28	28	19
minimal posterior distance between maxillae	46	40	40
foramen magnum-temporal crest	147	93	69
width lateral margins occipital condyles	107	88	78
height right occipital condyle	62	48	37
width foramen magnum	40	e39	35
height foramen magnum	42	e29	31
width posterior alveolus	12.5	—	3.7

The rostrum is less abruptly tapering from its base to its anterior end than in *Notocetus*, *Squalodelphis* and particularly *Huaridelphis*, all of them having a narrow anterior half and a wide, triangular posterior half of the rostrum. To better quantify this feature, also on skulls lacking the anterior portion of the rostrum, we measured the width of the rostrum at a distance from the rostrum base twice the width across the antorbital notches. The ratio between this measurement and the width at the antorbital notch is 0.37 in *Macrosqualodelphis* and <0.35 in *Huaridelphis*, *Notocetus* and *Squalodelphis*. A value close to the one of *Macrosqualodelphis* is observed in the aff. *H. raimondii* MUSM 603, also from Chilcatay Fm, as described by Lambert *et al.* [13].

The neurocranium is anteroposteriorly shorter than transversely wide, as in all other homodont platanistoids (*sensu* [13]).

As in the other squalodelphinids, the antorbital notches are distinctly asymmetrical, having (i) the right antorbital notch more posteriorly located than the left and (ii) the lateral and medial borders of the antorbital notch drawing a more open angle on the right side (86°) than on the left side (60°); the ratio between the two angles is approximately 1.4, intermediate between *Notocetus* (1.7) and *Huaridelphis* (1.2). Moreover, the left antorbital notch is more U-shaped than the V-shaped right antorbital notch. By contrast, in all other squalodelphinids, both antorbital notches are V-shaped.

As in all other squalodelphinids and in platanistids, the vertex and the bony nares are distinctly shifted on the left side, as clearly evidenced by the oblique orientation of the main transverse axis of the nasals and, perpendicular to this axis, of the nasal septum made of the presphenoid (see below).

**Figure 2.**
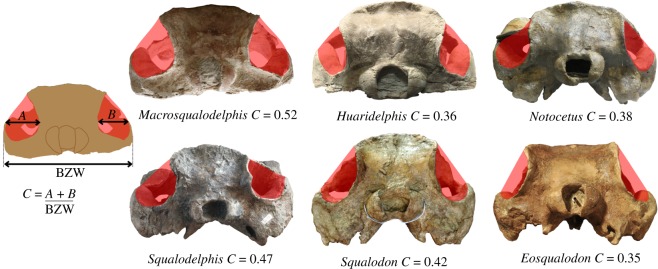
Comparison of the extent of temporal fossae in posterior view for *M. ukupachai* (MUSM 2545) and other squalodelphinids (*H. raimondii*, MUSM 1396; *N. vanbenedeni*, MLP 5-5; *Squalodelphis fabianii*, MGP 26134) and with two squalodontids (*Squalodon bellunensis*, MGP 26131; *Eosqualodon langewieschei*, BDNLTM 326). The crania are in posterior view and the cross-section of the temporal fossae is highlighted in red. The value of ‘*C*’ reported for each skull represents the ratio between the transverse width of the right + left temporal fossae and the BZW, as shown on the left of the figure. A higher value of *C* indicates temporal fossae proportionally transversely wider.

The temporal fossa is dorsoventrally higher and more anteroposteriorly elongated than in all other squalodelphinids, extending posteriorly beyond the occipital condyles due to a salient temporal crest. The temporal fossa also exhibits a significant transverse widening, as can be seen in posterior view. Among squalodelphinids, a similar widening is only observed in *Squalodelphis*, related to a more lateral position of the zygomatic process of the squamosal. It is interesting to note that, although squalodontids have a dorsoventrally and anteroposteriorly large temporal fossa large as in *Macrosqualodelphis*, their fossa is significantly transversely narrower than in *Macrosqualodelphis* (figure 2)*.*

#### 4.3.2. Premaxilla

Owing to the poor preservation of the anteroventral portion of the rostrum, the extent of the anterior premaxillary portion of the rostrum and the presence of dental alveoli in this apical premaxillary portion cannot be assessed.

**Figure 3.**
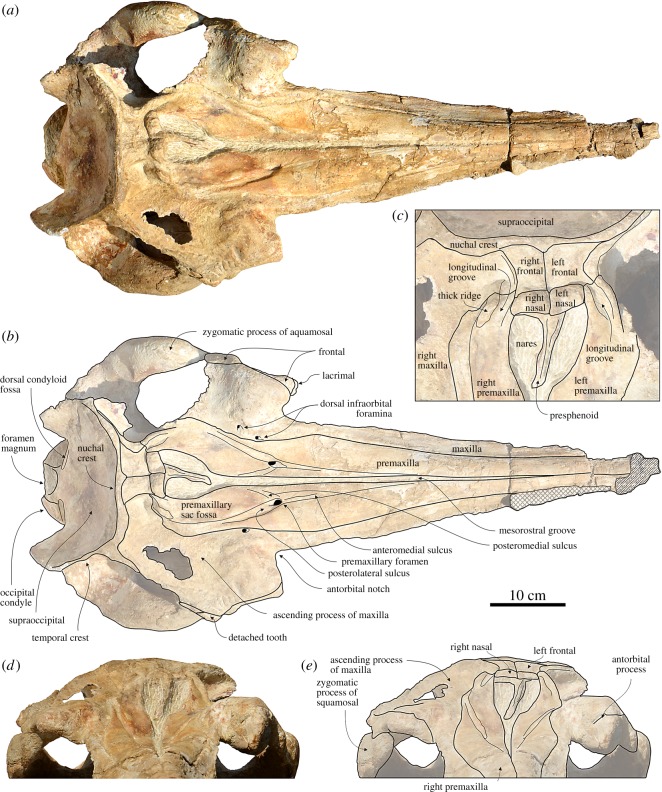
Cranium of the holotype (MUSM 2545) of *M. ukupachai*, from the early Burdigalian of the Chilcatay Fm (Pisco Basin, Peru). (*a*) Dorsal view; (*b*) corresponding explanatory line drawing; (*c*) detail of the vertex area in dorsal view; (*d*) anterior view; (*e*) corresponding explanatory line drawing. Linear hatching indicates major breaks and cross-hatching areas covered by the sediment.

In dorsal view, the medial margins of the right and left premaxillae contact each other for approximately 150 mm from the preserved anterior end of the rostrum; then the premaxillae gradually diverge towards the V-shaped bony nares (figure 3). The dorsal opening of the mesorostral groove remains narrow for all its anteroposterior extent, reaching a transverse width of 15 mm near the anterior end of the bony nares. This condition is intermediate between *Huaridelphis,* whose premaxillae contacting medially for about half the length of the rostrum, and *Notocetus*, whose mesorostral groove is open until or in close proximity of the apex of the rostrum. As in *Macrosqualodelphis*, both *Huaridelphis* and *Notocetus* retain a narrow opening of the mesorostral groove near the rostrum base, whereas *Medocinia* and *Squalodelphis* display a wide opening.

From the anterior end of the anteromedial sulcus, between 220 and 330 mm anterior to the right antorbital notch, the right premaxilla is clearly narrower than the left, a condition shared with all other squalodelphinids and most platanistids [13].

Both premaxillae exhibit their maximum transverse width approximately 60 mm anterior to the right antorbital notch, a condition also observed, with some degree of intraspecific variation, in the other squalodelphinids.

The posterior rostral portion of the premaxillae is also featured by a marked medial slope, forming a prenarial depression having its maximum depth at the level of the right antorbital notch. Here, the vertical distance between the lateral and the medial margins of the premaxilla reaches 18 mm. A similar prenarial depression is observed in all other squalodelphinids and, more or less marked, in most of the other homodont platanistoids [13].

A single premaxillary foramen is clearly visible on both premaxillae, at the level of the right anterior notch. Among the other squalodelphinids, the premaxillary foramen is anterior to the right antorbital notch in *Huaridelphis* and *Squalodelphis*, whereas its position varies from anterior to weakly posterior to the right antorbital notch in the three known skulls of *Notocetus*. The elongated anteromedial sulcus (approx. 15 mm) and the posteromedial sulcus are both weakly excavated, whereas the posterolateral sulcus is deep and clearly discernible, reaching posteriorly the anterior limit of the bony nares. The premaxillary sac fossa is narrow, weakly concave and slopes medially. The maximum transverse widths of the right and left premaxillary sac fossae are roughly identical. Both ascending processes of the premaxillae are deeply incised by a longitudinal groove laterally margined by a thick ridge. This groove might be homologous to the premaxillary cleft described in *Waipatia* [41] and also observed in *Papahu* [42]. It is also visible in all other squalodelphinids having this region well preserved and in most platanistids [13]. Right and left ascending processes of the premaxillae display a short posteromedial angle contacting the corresponding frontal at the vertex.

#### 4.3.3. Maxilla

In dorsal view, the transverse width of the maxilla is roughly constant on most of the length of the rostrum and consistently decreases at the level of the maximum widening of the premaxilla (at approx. 60 mm anterior to the right antorbital notch) (figure 3). At this level, the ratio between the transverse width of maxillae and the transverse width of the premaxillae reaches the minimum value of 0.30. More posteriorly, this ratio increases, reaching a value of 0.68 that is closer to *Huaridelphis* (0.60–0.61) and *Notocetus* (0.56–0.68), but significantly smaller than in *Medocinia* and *Squalodelphis* (0.82), both having the premaxilla nearly reaching the lateral margin of the rostrum.

In the posterior portion of the rostrum, the maxilla becomes dorsoventrally thinner laterally, with a slender, blade-like lateral margin of the rostrum, whereas this margin is significantly thicker in other squalodelphinids. This feature is clearly visible in lateral view, together with the steep ascent of this lateral margin towards the antorbital notch.

As in all other squalodelphinids with the exception of *Medocinia* (whose holotype skull only preserves the posterior part of the rostrum), the unfused lateral maxilla–premaxilla suture is not excavated by a deep groove, contrasting with most platanistids (except *Araeodelphis* [15], allodelphinids, eurhinodelphinids and eoplatanistids).

A single infraorbital foramen pierces the maxilla 50 mm posterior to the right antorbital notch, whereas two foramina are present 20 and 50 mm posterior to the left notch. All these foramina are located near the medial margin of the maxilla, in the area of the greatest concavity of the lateral margin of the premaxilla.

From the base of the rostrum, the maxilla extends posterolaterally, forming the posterior wall of the antorbital notch, but not covering the anterolateral portion of the preorbital process of the frontal and the antorbital process of the lacrimal. The antorbital process of the maxilla is elevated (the left more than the right) in relation to the dorsoventral thickening of both the maxilla and the frontal in this area. A similar thickening is also observed in other squalodelphinids: it is much more pronounced in *Squalodelphis*, almost absent in *Medocinia*, and similarly developed in *Notocetus*. An extreme condition is observed in platanistids, in which it forms an elevated maxillary (or frontomaxillary) crest. Posterior to the postorbital process, the maxilla and the underlying frontal become very thin and, consequently, they are broken and partially missing on the left side. On the right side, however, the lateral edge of the maxilla and underlying frontal is apparently preserved, although a large breakage is observed in the middle of the dorsal aspect of the maxilla. The posterior dorsal infraorbital foramina are apparently absent, but they could have been originally located on the missing part of the maxillae.

The left maxilla descends more abruptly ventrolaterally from the vertex than the right maxilla, forming a deep fossa posterolateral to the left nasal. Linked to the shift of the vertex towards the left side, this feature is also observed in other squalodelphinids, even if is less marked in *Huaridelphis*.

**Figure 4.**
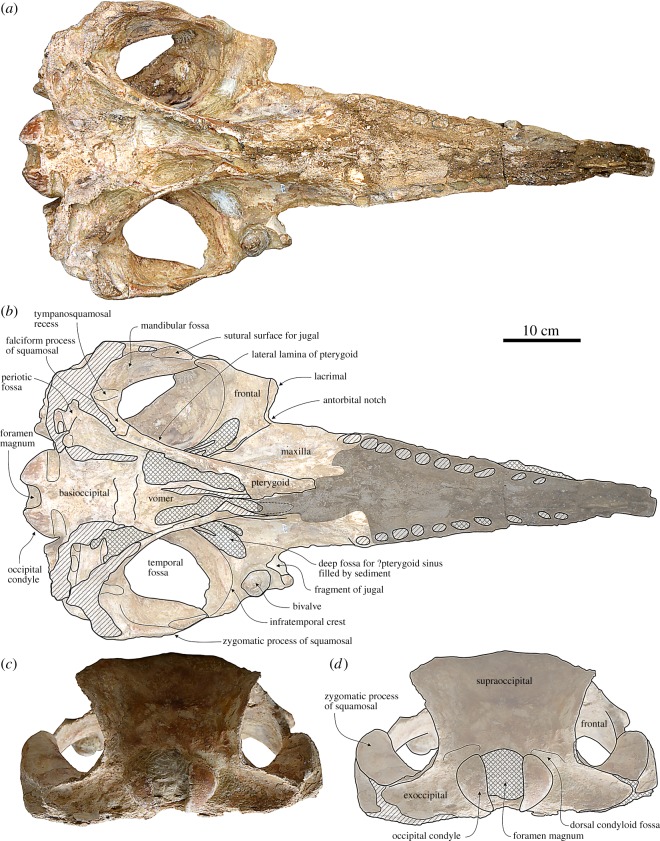
Cranium of the holotype (MUSM 2545) of *M. ukupachai*, from the early Burdigalian of the Chilcatay Fm (Pisco Basin, Peru). (*a*) Ventral view; (*b*) corresponding explanatory line drawing; (*c*) posterior view; (*d*) corresponding explanatory line drawing. Linear hatching indicates major breaks, cross-hatching areas covered by the sediment and dark shading worn surface.

**Figure 5.**
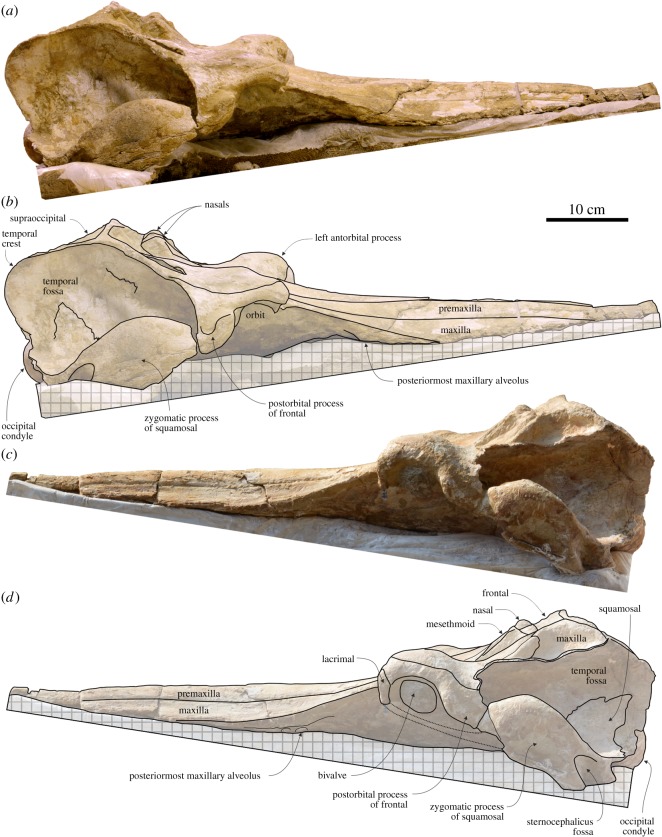
Cranium of the holotype (MUSM 2545) of *M. ukupachai*, from the early Burdigalian of the Chilcatay Fm (Pisco Basin, Peru). (*a*) Right lateral view; (*b*) corresponding explanatory line drawing; (*c*) left lateral view; (*d*) corresponding explanatory line drawing. Cross-hatching indicates supporting frame.

Owing to recent erosion, most of the palatal surface of the maxilla is missing (figure 4*a*,*b*). However, on the preserved portion of the rostrum, 10 eroded alveoli are visible near the lateral margin of each maxilla. Most of these alveoli still hold partly broken single-rooted teeth. The transverse diameter of the alveoli ranges from 10 mm anteriorly to 13 mm posteriorly. The apparent smaller transverse diameter of the anterior alveoli is due to the fact that the cross-sections of the alveoli and associated dental roots are closer to their narrower deeper portions. Although the spacing of the alveoli varies irregularly along the alveolar row, it is smaller posteriorly (0–5 mm) than anteriorly (up to 20 mm). The posteriormost right alveolus is 106 mm anterior to the right antorbital notch (115 mm on the left side). Posterior to the posteriormost alveolus, the maxilla rises abruptly posterodorsolaterally, generating a deep excavation of the ventral surface of the rostrum near its lateral margin, a feature clearly visible in ventral and lateral view (figures 4*a*,*b* and 5). This excavation is related to the above-mentioned thin lateral margin of the posterior part of the rostrum.

#### 4.3.4. Presphenoid

The ossified portion of the presphenoid ( = mesethmoid from previous works; see [43]) exhibits a narrow and elongated nasal septum separating the bony nares (figure 3). This septum draws an angle of 14° with the main axis of the skull. The posterodorsal margin of the cribriform plate reaches the anterodorsal margin of the nasals.

#### 4.3.5. Nasal

The nasals are nodular, with an inflated and subhorizontal dorsal surface, reaching a level higher than the frontals (figures 3 and 5). For all these features, the nasals of *Macrosqualodelphis* are similar to those of *Notocetus*, whereas they differ from those of the other squalodelphinids, all characterized by a flat and anteriorly sloping dorsal surface of the nasals. Nevertheless, the nasals of *Macrosqualodelphis* differ from those of *Notocetus* in their smaller general size and in being proportionally anteroposteriorly shorter.

There is no significant difference in size and shape between the right and left nasals. The longitudinal axis of the nasals is slightly obliquely oriented, drawing an angle of 6° with the main axis of the skull, and the anterolateral corner of the right nasal is 10 mm anterior to the anterolateral corner of the left nasal. A similar oblique orientation of the longitudinal axis of the nasals is present in *Huaridelphis*, *Notocetus* and *Squalodelphis*. The anterior margin of both nasals is weakly anteriorly convex, with the anterior edge of the joined nasals forming an anteromedial angle of 155°. The nasal-frontal suture is on the whole straight, with only a small anteromedial process of the left frontal wedged between the nasals. The condition of *Macrosqualodelphis* is intermediate between *Notocetus*, whose anterior and posterior margins of the nasals are anteriorly convex, and *Huaridelphis*, whose both margins are straight.

#### 4.3.6. Frontal

The dorsal exposure of both frontals at the vertex slopes anteroventrally and has a minimal transverse width slightly smaller than the transverse width of nasals (figure 3). The right and left frontals are subequal in length, differing in this respect from *Huaridelphis*, *Notocetus* and *Squalodelphis*, but resembling *Medocinia.* By contrast, the left frontal is significantly transversely wider than the right as observed in the other squalodelphinids (but the medial suture between the frontals is not visible in *Squalodelphis*). The medial suture between the frontals is straight and the frontal–occipital suture is transversely oriented with a small anteromedial process of the supraoccipital wedged between the frontals anteriorly.

The preorbital process of the frontal is dorsoventrally thickened (more than in *Huaridelphis* and less than in *Medocinia*), the orbit is anteroposteriorly short, and the postorbital process is robust and dorsoventrally elongated (figure 5). On the medial portion of the ventral surface of the orbit roof, a fossa is partially filled by sediment (figure 4*a*,*b*). A similar fossa, but slightly larger and deeper, has been observed in *Huaridelphis* and *Notocetus*, and interpreted as corresponding to an extension of the pterygoid sinus in the orbit region [13,44].

#### 4.3.7. Supraoccipital

The nuchal crest is prominent and, unlike in all other squalodelphinids, higher than the frontals and the nasals at the vertex (figures 3*a–c* and 5). This crest is markedly transversely wide, as in all other squalodelphinids with the exception of *Squalodelphis*, the latter having a narrower nuchal crest (approximately as wide as the greatest width of the premaxillae). The nuchal crest of *Macrosqualodelphis* is roughly straight in dorsal view, whereas the outline of the supraoccipital shield, formed medially by the nuchal crests and laterally by the two prominent temporal crests, is half-circle shaped in posterodorsal view.

In dorsal view, the temporal crest extends far posterolaterally, increasing the length of the temporal fossa. The complete right temporal crest extends far beyond the occipital condyles.

The posterodorsal surface of the supraoccipital shield is transversely concave and there is no external occipital crest (*sensu* [25]) (figure 4*c*,*d*).

#### 4.3.8. Palatine

The palatines cannot be identified on the ventral surface of the skull, either due to their complete fusion with the maxillae or because they are fully covered by the pterygoids (figure 4*a*,*b*).

#### 4.3.9. Pterygoid

The pterygoid is long and narrow, as in other squalodelphinids and platanistids. Its pointed anterior apex extends 60 mm beyond the level of the right antorbital notch (figure 4*a*,*b*).

The pterygoid sinus fossa reaches the level of the right antorbital notch, whereas it extends beyond the antorbital notch in other squalodelphinids.

The lateral lamina of the pterygoid is a robust, plane plate that contacts posteriorly the falciform process of the squamosal as is observed in all the other squalodelphinids.

#### 4.3.10. Jugal–lacrimal

In ventral view, the lacrimal and the fused preserved anterior portion of the jugal are longitudinally short as in the other squalodelphinids (figure 4*a*,*b*). They form the anteriormost portion of the ventral surface of the antorbital process and, more medially, the posterior margin of the antorbital notch.

A narrow ventral projection of the lacrimojugal complex is preserved on the left side of the skull (figure 5*c*,*d*). A similar peculiar structure was also observed in the holotype of *N. vanbenedeni* and seems to be analogous to the ventroposterior projection of the jugal described in the holotype of the eurhinodelphinid *Eurhinodelphis cocheteuxi* du Bus, 1867 [45] by Lambert ([46], fig. 3).

#### 4.3.11. Squamosal

In lateral view, the zygomatic process of the squamosal shares with the other squalodelphinids the same strongly swollen aspect (figure 5). This process is more anteriorly elongated than in *Huaridelphis*. Its posterodorsal margin is markedly convex and its anteroventral margin is slightly convex. The ratio between the maximum distance from the anteroventral margin of the zygomatic process to its posterodorsal margin, in lateral view, and the vertical distance from the lower margin of the occipital condyles to the vertex of the skull is 0.43, a value indicating a robustness of the zygomatic process similar to other squalodelphinids.

As mentioned above, the postglenoid process is lacking in both squamosals, due to recent erosion of the skull.

In ventral view, a deep, narrow, 50 mm long depression on the anterolateral margin of the zygomatic process represents the suture for the missing posterior portion of the jugal. The mandibular fossa is wide, occupying most of the ventromedial surface of the zygomatic process and being laterally defined by the thin ventral margin of the process. The tympanosquamosal recess is transversely narrow. More medially, the falciform process is a wide plate contacting anteriorly the lateral lamina of the pterygoid.

#### 4.3.12. Exoccipital

The occipital condyles are posteriorly prominent with a conspicuous neck in ventral view (figure 4*c*,*d*). The dorsal condyloid fossae are visible in posterior view, dorsolateral to the occipital condyles. The exoccipital extends far laterally and its dorsal margin contributes significantly to the transversely wide posteroventral margin of the temporal fossa. Together with all parts of the exoccipital ventral to the condyles, the paroccipital processes are missing.

#### 4.3.13. Basioccipital

The ventral surface of the basioccipital is not well preserved and the basioccipital crests are broken and almost completely missing (figure 4*a*,*b*).

#### 4.3.14. Vomer

In ventral view, the vomer is visible between the choanae, in a region partly filled with sediment (figure 4*a*,*b*).

### 4.4. Teeth

Based on the abraded ventral surface of the rostrum showing broken dental roots and alveoli, each maxilla carried more than 10 single-rooted teeth (figure 4*a*,*b*). Moreover, considering the estimated missing portion of the rostrum (154 mm), it is probable that the original upper tooth count per quadrant reached a value close to *Squalodelphis* (15) and lower than other squalodelphinids for which the upper tooth count is known (*Huaridelphis*, 28–30; *Notocetus*, 22–23).

The posterior maxillary alveoli have a transverse diameter of approximately 13 mm, that is 3.5% of the BZW, a value higher than in other squalodelphinids (all with values lower than 3.0%).

**Figure 6.**
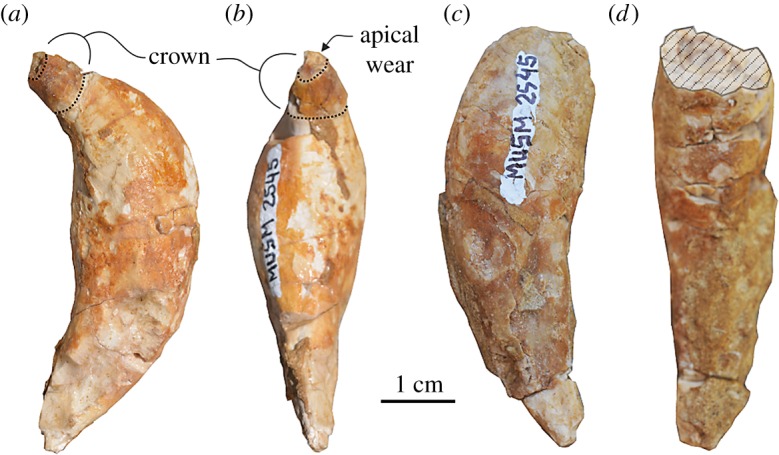
Detached teeth of the holotype (MUSM 2545) of *M. ukupachai*, from the early Burdigalian of the Chilcatay Fm (Pisco Basin, Peru). (*a*,*b*) Complete tooth; (*c*,*d*) broken large tooth with crown missing. Linear hatching indicates major breaks.

Only one complete and two fragmentary detached single-rooted teeth are preserved (figure 6). The complete tooth, the only one having the crown preserved, is 55 mm in length. It is curved and crescentiform in labial and lingual views, and straight and fusiform in mesial and distal views. The crown is small, having a diameter at its base of only 8 mm contra 48 mm of the maximum diameter of the root. The root is transversely flattened (ratio between the maximum mesiodistal and labiolingual diameters of 0.33). The maximum transverse diameter of the root is 18 mm, contrasting with the transverse diameter of the posterior maxillary alveoli reaching roughly 13 mm, suggesting that anterior teeth were significantly larger than the posterior teeth. The two other teeth only preserve the root, with a length of 58 and 55 mm corresponding to teeth even bigger than the complete tooth described above. Considering their shape and large size, with a diameter significantly larger than the posterior maxillary alveoli, it is probable that these teeth originate from the missing anterior portions of the rostrum and of the mandibles. Similar large anterior teeth are also present in *Squalodelphis*, and several platanistids including the extant *Platanista*, which displays anterior teeth considerably larger than posterior ones [47,48].

### 4.5. Vertebrae

#### 4.5.1. Atlas

**Figure 7.**
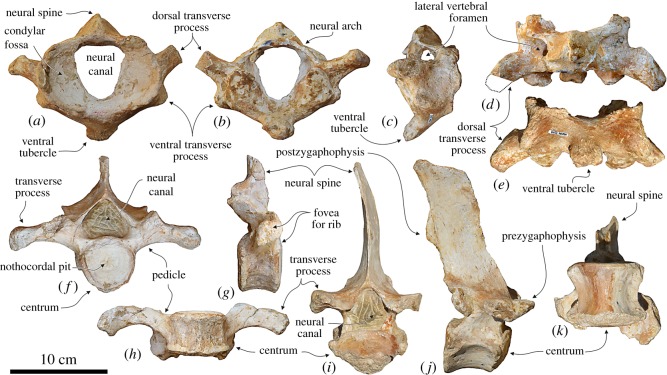
Cervical and thoracic vertebrae of the holotype (MUSM 2545) of *M. ukupachai*, from the early Burdigalian of the Chilcatay Fm (Pisco Basin, Peru). (*a–e*) Atlas in anterior (*a*), posterior (*b*), right lateral (*c*), dorsal (*d*) and ventral (*e*) views; (*f–h*) thoracic (T1?) in posterior (*f*), right lateral (*g*) and ventral (*h*) views; (*i–k*) thoracic (T4 or T5) in posterior (*i*), right lateral (*j*) and ventral (*k*) views.

The well-preserved, anteroposteriorly thick atlas of *Macrosqualodelphis* is not fused to the missing axis (figure 7*a–e* and table 2). It is similar to the atlas of *Notocetus* [49] in having: (i) elongated and dorsoposteriorly projected dorsal transverse process; (ii) short ventral transverse process; (iii) broad and posteriorly projected ventral tubercle; (iv) low neural arch; and (v) short and broad neural spine. The only substantial difference is the shape of the neural canal: transversely compressed in *Macrosqualodelphis* (ratio between width and height = 0.76) and roughly circular in *Notocetus* (ratio = 1.07, using measurements of True [49]). The lateral vertebral foramen is very large and opens laterally near the base of the neural arch.

**Table 2.** Measurements of the cervical (C), thoracic (T), lumbar (L) and caudal (Ca) vertebrae of *M. ukupachai* holotype (MUSM 2545). Width of the vertebra includes the transverse processes. Height of the vertebra includes the neural spine. Centra are measured on the anterior surface. All measurements are in mm. +, incomplete; —, missing data; e, estimate.

	C1	T1	T5–6	Ln-1	Ln	Ca1	Ca2	Ca3	Ca4	Cax-2	Cax-1	Cax
width of vertebra	e180	200	e114	e256	e280	e260	e266	e250	e192	55	47	41
height of vertebra	115	146	220	+273	+250	+235	+210	—	—	41	37	32
centrum length	—	44	66	108	111	112	102	95	92	24	20	22
centrum width	—	59	66	85	87	88	92	90	86	46	40	34
centrum height	—	58	55	85	87	86	84	84	84	37	35	28
neural canal width	41	50	42	18	12	12	12	12	—	—	—	—
neural canal height	54	41	40	30	27	+23	26	20	—	—	—	—

#### 4.5.2. Thoracic vertebrae

One of the two preserved thoracic vertebrae closely resembles the vertebra of *Notocetus* described as the first thoracic (T1) ([49], pl. 5, figs 3–4), having an anteroposteriorly short centrum (ratio between length and height of the centrum = 0.7) and elongated transverse processes slightly ventrolaterally directed (figure 7*f–h*). Such elongated transverse processes are not observed in the ‘Thoracic A’ (possible T1) of *Huaridelphis* ([13], fig. 5Q,R) and in the T1 of *Phocageneus venustus* Leidy, 1869 [50] USNM 21039 [51]. Nevertheless, the vertebra of *Macrosqualodelphis* differs from the T1 of *Notocetus* in the more robust transverse processes, the roughly circular outline of the centrum in anterior and posterior views (heart-shaped in *Notocetus*) and the more transversely compressed neural arch.

The other preserved thoracic vertebra of *Macrosqualodelphis* exhibits a more anteroposteriorly elongated centrum (ratio between length and height of the centrum = 1.2), shorter transverse processes, a dorsoventrally compressed, heart-shaped outline of the centrum in anterior and posterior views, pedicles vertically rather than obliquely oriented, a narrower neural arch and a higher neural spine (figure 7*i–k*). This vertebra is very similar to the ‘Thoracic D’ of *Huaridelphis*, also sharing similarities with T4–T5 of *Phocageneus venustus*. These latter differ from the vertebrae of *Macrosqualodelphis* and ‘Thoracic D’ of *Huaridelphis* in having a more rounded ventral margin of the centrum in anterior and posterior views and a neural spine vertical rather than posteriorly inclined.

#### 4.5.3. Lumbar vertebrae

**Figure 8.**
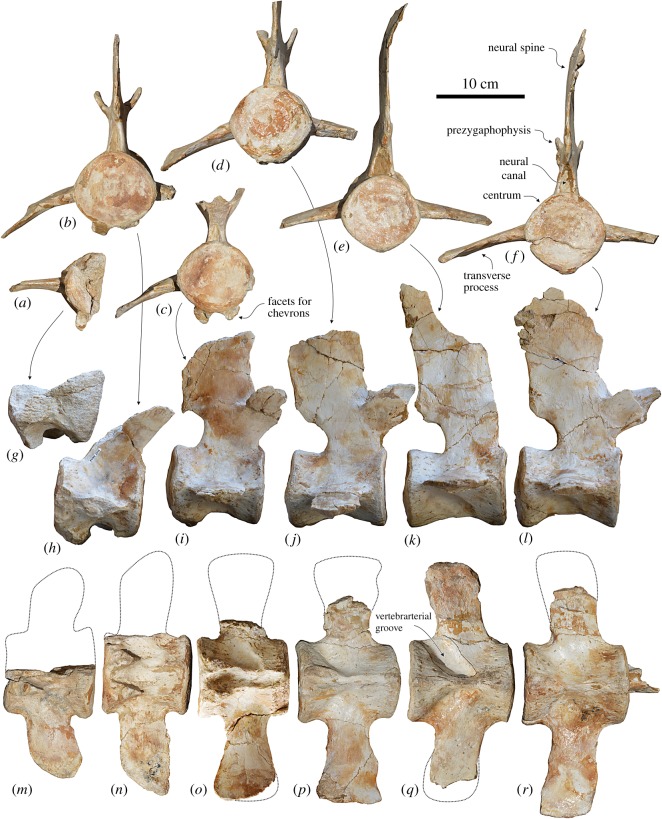
Lumbar and caudal vertebrae of the holotype (MUSM 2545) of *M. ukupachai*, from the early Burdigalian of the Chilcatay Fm (Pisco Basin, Peru). (*a*,*g*,*m*) Ca4; (*b*,*h*,*n*) Ca3; (*c*,*i*,*o*) Ca2; (*d*,*j*,*p*) Ca1; (*e*,*k*,*q*) last lumbar; (*f*,*l*,*r*) penultimate lumbar; in posterior (*a–f*), right lateral (*g–l*) and ventral (*m–r*) views.

**Figure 9.**
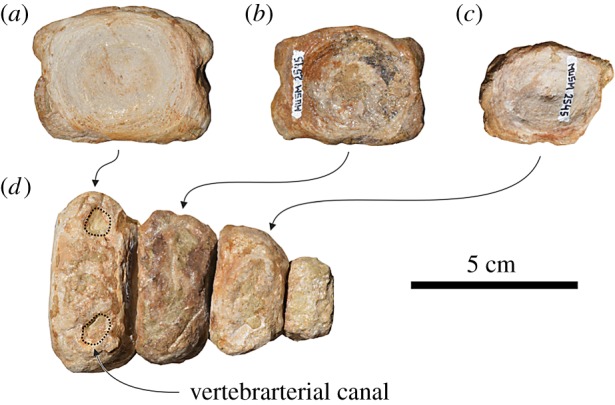
Last caudal vertebrae of the holotype (MUSM 2545) of *M. ukupachai*, from the early Burdigalian of the Chilcatay Fm (Pisco Basin, Peru). (*a–c*) Anterior views; (*d*) ventral view.

Two large vertebrae are interpreted as the two last lumbars, because they are the anteriormost and the only vertebrae without facets for the chevrons (haemal arches) of a sequence of six vertebrae found in articulation (figure 8*e*,*f*,*k*,*l*,*q*,*r*). These two vertebrae have a cylindrical, elongated centrum (ratio between length and height of the centrum = 1.26–1.27), bearing a marked medial keel on the ventral surface. A pair of wide and deep sulci runs obliquely from the centre of the ventral surface forward with the posterolateral margins. Similar grooves are also present on the following Ca1–Ca4 of MUSM 2545 (figure 8*m–p*) and have been named ‘hypovertebral grooves’ by Aguirre-Fernández *et al.* ([52], fig. 9) on two isolated lumbar vertebrae from the Miocene of Venezuela. According to these authors, together with the proportionally very elongated centrum, the presence of hypovertebral grooves supports the assignation of the two vertebrae from Venezuela to cf. *Zarhachis flagellator* Cope, 1868 [53], because similar grooves have been first observed on the four vertebrae described as the type material of *Z. flagellator* [53,54]. However, similar grooves have been described in several cetaceans and interpreted as related to the passage of the arteries departing from the caudal portion of the abdominal aorta [55]. We observed the same grooves, although generally less excavated than in *Macrosqualodelphis*, on the lumbar and caudal vertebrae of most of the extant and many fossil odontocetes. In some cases, on the caudal vertebrae, we note that each groove is laterally connected to the vertebrarterial canal, suggesting, as already pointed out by Slijper [55], that the artery runs along the groove and crosses the transverse process of the vertebra to reach the dorsal tissues. We therefore rather use the term ‘vertebrarterial groove’ instead of ‘hypovertebral groove’ as proposed by Aguirre-Fernandez *et al.* [52]. Furthermore, by denying any systematic relevance to this character, we suggest that the referral to the family Platanistidae of the lumbar vertebrae from the Miocene of Venezuela should remain tentative.

The elongated transverse processes of the two lumbar vertebrae of *Macrosqualodelphis* start from the lateral borders of the centrum, and are dorsoventrally flattened, weakly widened distally and ventrally and posteriorly directed. The neural canal is narrow and high. Partly preserved only on the anteriormost vertebrae, the prezygapophyses are large and dorsolaterally inclined. The neural spine is anteroposteriorly wide and slightly posteriorly inclined.

#### 4.5.4. Caudal vertebrae

Four of the eight preserved caudal vertebrae are presumably the anteriormost ones (Ca1–Ca4) (figure 8*a–e*,*g–j*,*m–p*). They are close in size and shape to the posteriormost lumbars, the main difference with the lumbars being the presence of facets for the chevrons. Other differences are the lesser elongation of the centrum (slightly decreasing from Ca1 to Ca4), the smaller size of the neural arch and the transverse processes being more posteriorly directed, but perpendicular to the longitudinal axis of the centrum in dorsal and ventral views. Moreover, the transverse processes of Ca3 and Ca4 do not widen distally, being instead anteroposteriorly pointed.

The other four preserved caudal vertebrae are probably the last ones, corresponding to the fluke region (figure 9). They are considerably smaller compared to Ca1–Ca4, they lack transverse processes and neural arch, and are anteroposteriorly and dorsoventrally compressed. Their surface is damaged by erosion and the vertebrarterial canals are only partly visible on the dorsal and ventral surface. The smaller last caudal has an irregular nodular shape.

### 4.6. Forelimb

**Figure 10.**
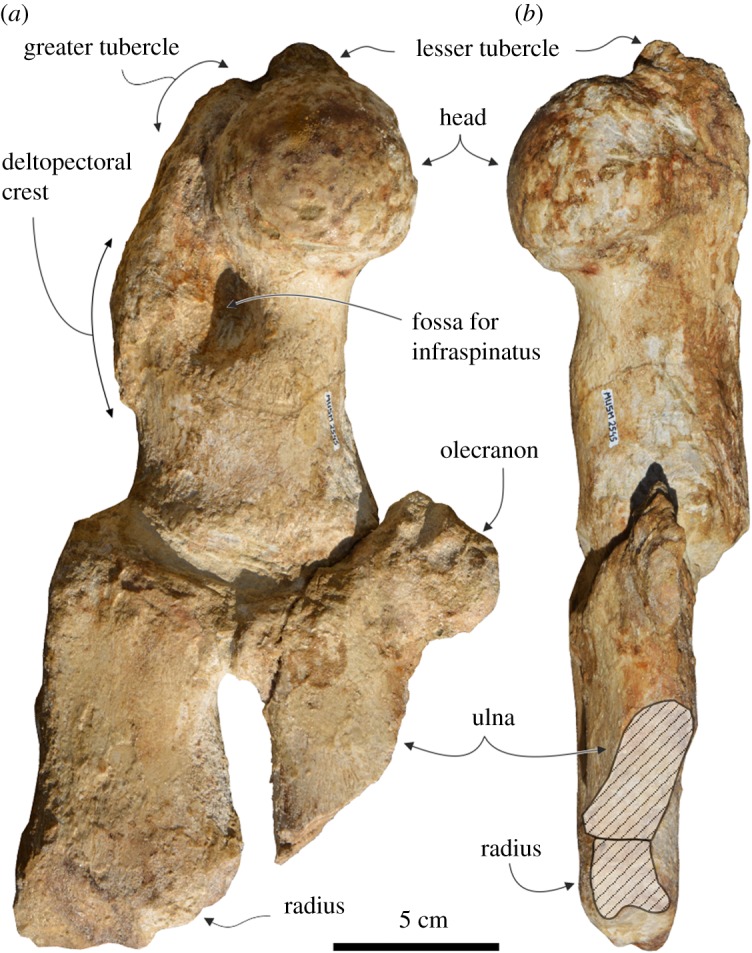
Left humerus, radius and ulna in anatomical connection of the holotype (MUSM 2545) of *M. ukupachai*, from the early Burdigalian of the Chilcatay Fm (Pisco Basin, Peru). (*a*) Lateral view; (*b*) posterior view. Linear hatching indicates major breaks.

The humerus, radius and ulna of the left forelimb have been maintained in anatomical connection after preparation (figure 10), as found in the field, whereas the two manus bones were found scattered in the sediment.

#### 4.6.1. Humerus

The humerus is robust and transversely flattened, stockier than in allodelphinids and waipatiids [56–58]. It is somewhat longer than the radius (ratio between their respective lengths = 1.24). The humerus is similarly longer than the radius in only a few extant odontocetes, including monodontids, physeteroids, *Inia* and *Platanista* [59,60], whereas this feature is commonly observed in extinct platanistoids and related taxa (e.g. allodelphinids, eurhinodelphinids, the early platanistoid *Otekaikea huata*, the squalodontid *Kelloggia* (probably synonymous to *Squalodon*) *barbara* Mchedlidze, 1976 [56] and the probable waipatiid *Sulakocetus* [5,57]). The humeral head is hemispherical and protrudes posterolaterally. Medially to the head, the lesser tubercle is robust, higher than the head and the smaller, anteriorly located greater tubercle. The greater tubercle lies on the anteromedial margin of the humerus, extending distally in a salient and elongated deltopectoral crest. This crest reaches a level closer to the distal epiphysis than observed in allodelphinids, *Otekaikea huata* Tanaka & Fordyce, 2015 [5], and waipatiids, whereas *Platanista* lacks any defined crest [47,59]. On the lateral surface of the diaphysis, posterior to the deltopectoral crest, there is a large and deep fossa for insertion of M. infraspinatus. The posterior margin of the humerus is concave, due to the slight anteroposterior widening of the distal epiphysis (to a much lesser extent than in *Platanista*).

#### 4.6.2. Radius

The radius lacks the posterior portion of its distal epiphysis. It is a transversely flattened trapezoidal bone that slightly widens distally. It is proportionally longer than in *Platanista*, but considerably shorter, stockier than in allodelphinids, *O. huata* and waipatiids, and more similar to some eurhinodelphinids (e.g. *Schizodelphis* sp. USNM 244413) and squalodontids (e.g. *Kelloggia barbara* [56] and *Squalodon bellunensis* Dal Piaz, 1901 [61] MGP 26092). The radius is proximally articulated with the humerus and, for a small tract of its posterior margin, with the ulna.

#### 4.6.3. Ulna

The ulna lacks almost its whole distal half. Like the radius, it is transversely flattened; it is strongly anteriorly articulated with the latter bone and proximally with the humerus. The olecranon is roughly half-circle shaped in lateral and medial view, forming an open notch with the posterior margin of the diaphysis. The olecranon is less developed anteroposteriorly than in allodelphinids and waipatiids, with proportions more similar to eurhinodelphinids and squalodontids, whereas *Platanista* lacks such a process. Distal to the olecranon, the ulna is significantly anteroposteriorly narrower than the radius.

#### 4.6.4. Manus bones

**Figure 11.**
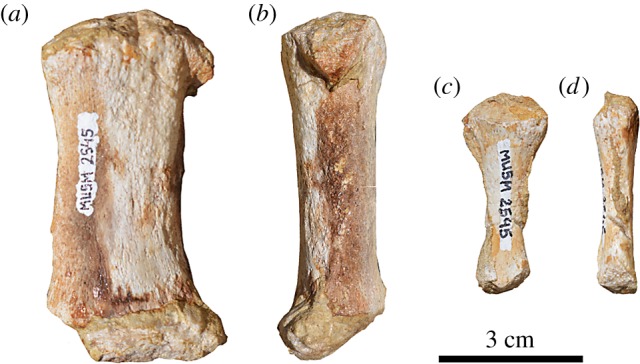
Manus bones of the holotype (MUSM 2545) of *M. ukupachai*, from the early Burdigalian of the Chilcatay Fm (Pisco Basin, Peru). (*a*,*b*) Metacarpal; (*c*,*d*) phalanx; in lateral (*a*,*c*) and anterior or posterior (*b*,*d*) views.

The preserved bones of the manus are two transversely flattened and straight small bones that differ significantly one from the other in the size and shape (figure 11). The largest has the mesial and distal epiphyses wider than the diaphysis. Owing to its large size and convex proximal margin in lateral and medial view, this bone occupied a more proximal position along the corresponding digit, probably as a metacarpal.

The smaller bone narrows significantly distally and is interpreted as a phalanx, located in a distal position along the corresponding digit.

These two bones do not differ significantly from the manus bones of *Platanista* and *Z. flagellator* [47,54,59].

## 5. Phylogeny

**Figure 12.**
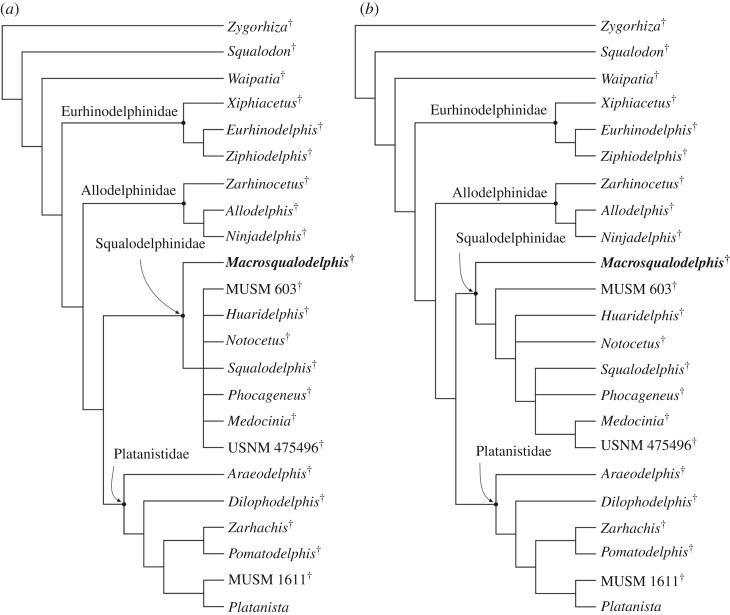
Result of the main phylogenetic analysis showing the relationships of *M. ukupachai* with the other nearly homodont Platanistoidea*.* (*a*) Consensus tree of 120 equally parsimonious trees, with tree length = 80, consistency index (CI) = 0.60 and retention index (RI) = 0.82. (*b*) 50% majority-rule consensus tree.

The cladistic analysis produced 120 equally parsimonious trees, with tree length = 80, consistency index (CI) = 0.60 and retention index (RI) = 0.82. The strict consensus tree and the 50% majority-rule consensus tree are presented in figure 12.

The strict consensus tree obtained here shows the same relationships within the homodont platanistoids as the tree of Godfrey *et al.* [15], summarized in the basalmost position of Allodelphinidae and the sister group relationship between Platanistidae and Squalodelphinidae, both families resulting as monophyletic groups. This analysis also confirms the position of *Dilophodelphis* within the platanistids, as already proposed by Boersma *et al.* [44] using a matrix modified from Godfrey *et al.* [15].

Our consensus tree supports the referral of *Macrosqualodelphis* to the family Squalodelphinidae, of which it is the earliest diverging lineage. The relationships between other squalodelphinids remain unresolved, as in previous analyses [13,15,44]. The 50% majority-rule consensus tree provides a more satisfactory result, with the specimen MUSM 603 branching before the two other South American genera *Huaridelphis* and *Notocetus* (unresolved relationships), and a clade including all the squalodelphinids from the North Atlantic realm.

The referral of *Macrosqualodelphis* to the family Squalodelphinidae is also supported when this taxon and *Huaridelphis* are included in the taxonomically broader matrix of Tanaka & Fordyce [6], as modified by Lambert *et al.* [20] and with the few further changes and additions reported in the Material and methods section and in table 4 of appendix B. Analysis 1 (equally weighted characters and no molecular constraint) produced 3919 equally parsimonious trees, with tree length = 1839, CI = 0.24 and RI = 0.65; analysis 2 (down-weighted homoplastic characters and no molecular constraint) produced 189 equally parsimonious trees, with tree length = 1888, CI = 0.23 and RI = 0.64; analysis 3 (equally weighted homoplastic characters and molecular constraint) and analysis 4 (weighted homoplastic characters and molecular constraint) both produced 272 equally parsimonious trees with tree length = 1925, CI = 0.23 and RI = 0.63 (consensus trees in figure 17 of appendix B).

Although this second set of analyses also supports the monophyly of Squalodelphinidae and the sister group relationship between Platanistidae and Squalodelphinidae, it does not resolve the relationships within the squalodelphinids.

## 6. Biostratigraphic and ^40^Ar/^39^Ar age constraint for *Macrosqualodelphis ukupachai*

The age of the Chilcatay Fm has been described in the past literature as spanning from the latest Oligocene to the earliest Middle Miocene based on diatoms, foraminifers and molluscs [34–37]. In the Western Ica Valley area, our biostratigraphic and ^40^Ar/^39^Ar datings converge and constrain the age of this formation to the Early Miocene.

In Roca Negra, the type locality of the heterodont dolphin *Inticetus vertizi* Lambert *et al*. [20], the base of the Chilcatay Fm was assigned through silicoflagellate biostratigraphy to the *Naviculopsis ponticula* zone of Bukry [62], dated by Bukry [63] between 19 and 18 Ma by correlation with the coccolith *Sphenolithus belemnos* zone at DSDP Site 495 offshore Guatemala [20]. We identified the same biozone at the locality of Ullujaya, a rich fossil marine vertebrate-bearing locality [13,19,21]. In the latter locality, the presence of *N. ponticula* subsp. *spinosa* indicates, following the species dominance described by Bukry [64], a slightly younger age within the same biozone.

**Figure 13.**
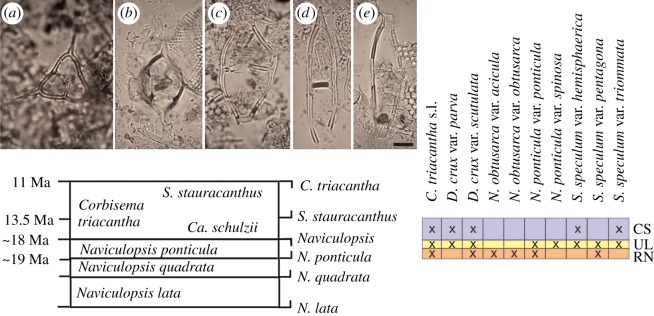
Silicoflagellate biostratigraphic scheme (redrawn after Perch-Nielsen [65]), stratigraphic range chart of silicoflagellates occurring at the different Chilcatay Fm localities of the Western Ica Valley area: RN, Roca Negra; UL, Ullujaya; CS, Cerro Submarino, and microphotographs of selected silicoflagellate species from RN: (*a*) *C. triacantha*; (*b*) *D. crux* subsp. *parva*; (*c*) *Naviculopsis obtusarca* var. *obtusarca*; (*d*) *N. obtusarca* var. *acicula*; (*e*) *N. ponticula* subsp. *ponticula*. Scale bar, 10 µm.

The top of the Chilcatay Fm is constrained at 18.02 ± 0.07 Ma, through ^40^Ar/^39^Ar age dating of a tephra layer collected by us 1 m below the erosional contact with the overlying Pisco Formation at Cerro Submarino. At the same locality, within diatomaceous sediments, the presence of *Corbisema triacantha*, *Distephanopsis crux* subsp. *parva* and subsp. *scutulata*, *Stephanocha speculum* cf. *triommata* and the absence of *Naviculopsis* allows us to assign these samples to the *Cannopilus schulzii* subzone within the *C. triacantha* zone, dated between 18 and 13.5 Ma [63] (figure 13).

The tephra layer CHILC-AT1, sampled 1.7 km SE of the holotype of *M. ukupachai*, near an uncollected squalodelphinid skeleton most likely belonging to the same taxon, is composed of 90% glass shards and 10% juvenile crystals, mainly biotite, as estimated by optical microscopy. EPMA analyses on volcanic glasses show a rhyolitic composition, whereas the biotite crystals suggest a calc-alkaline origin. Biotite analyses reveal a slight loss of K in the interlayer occupancy, but the petrological composition, the chemical homogeneity of the biotite population and the lack of sedimentary evidence suggest that this tephra layer is a primary air-fall. The level of post-eruptive marine alteration was low.

**Figure 14.**
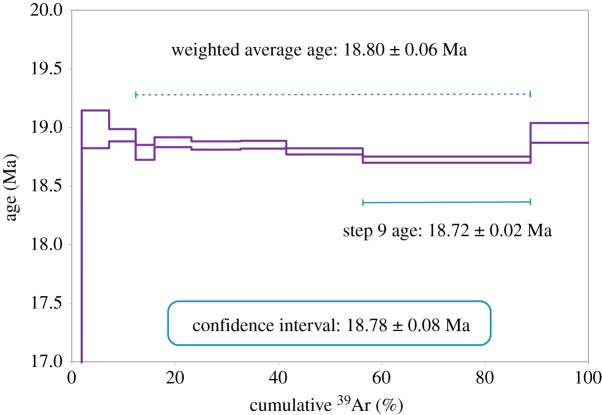
^40^Ar/^39^Ar age spectrum of the biotite separate of the tephra layer CHILC-AT1. The most conservative age assignment, taking into account interpretive ambiguities at the 0.4% level, is 18.78 ± 0.08 Ma. All uncertainties are shown as 2*σ*.

Considering the ‘isochemical steps’ [66] as the heating steps most closely reflecting the degassing of biotite crystals, we calculated the ^40^Ar/^39^Ar age from steps 4–9 with the lowest Ca/K and Cl/K ratios, obtaining a weighted average of 18.80 ± 0.06 Ma (2*σ*), with a mean square weighted deviation (MSWD) value of 12, and an isochron age of 18.70 ± 0.13 Ma (2*σ*), with an MSWD value of 7.3 (figure 14 and appendix C). However, both these dispersion values are too high, which points to a systematic bias, such as suggested by the substoichiometric K concentration of 6.8% calculated from the total ^39^Ar release. For this reason, we can consider only the age given by step 9, which is the most gas-rich step (greater than 30%) and the one with the lowest Ca/K: the age calculated is 18.72 ± 0.02 Ma (2*σ*), which overlaps with the weighted average, as shown in figure 14.

The most conservative age estimate covers the entire 2-sigma confidence interval between 18.70 and 18.86 Ma. If this confidence interval was symmetrical and Gaussian, it would correspond to an age of 18.78 ± 0.08 Ma, which can be considered as the age of this tephra layer. The Ar results are available in appendix C.

## 7. Discussion

### 7.1. Body size

**Figure 15.**
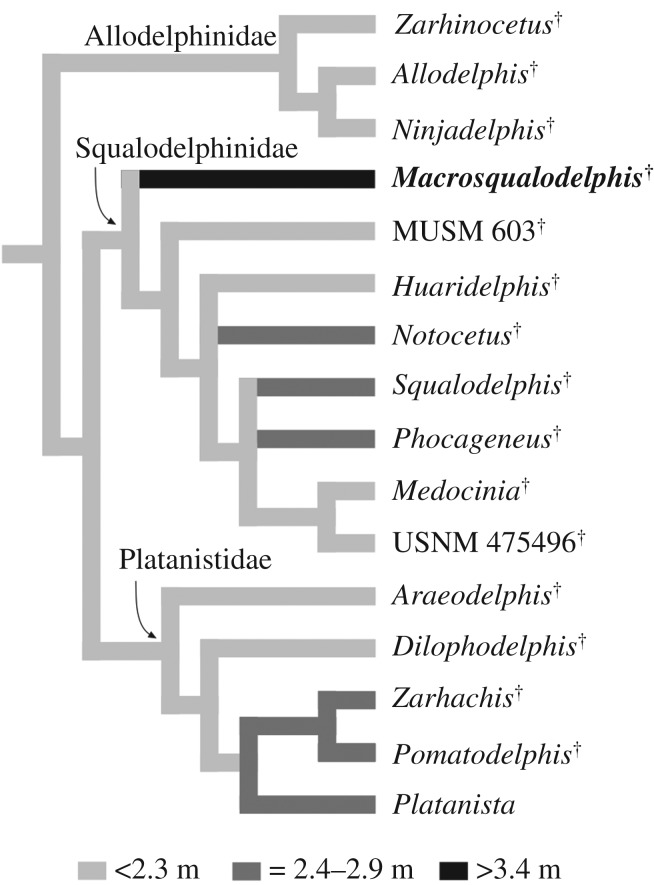
Evolution of body size among nearly homodont Platanistoidea. The result was obtained using Mesquite 2.74 [31], with the 50% majority-rule consensus tree as a backbone. Values for each genus correspond to the maximum size recorded. TBLs of the fossil taxa were estimated using the equation in Pyenson & Sponberg [30] for stem Platanistoidea. For the extant *P. gangetica*, we considered the maximum body sizes (2.6 m) reported for the adult females in Jefferson *et al.* [39]. Note that with its estimated TBL of 3.5 m, *Macrosqualodelphis* is markedly larger than any other homodont platanistoid analysed here.

Changes in the TBL of all platanistoids with single-rooted posterior teeth included in our phylogenetic analysis have been investigated as reported in the Material and methods section. The results confirm that with an estimated TBL of 3.5 m *Macrosqualodelphis* is by far the largest homodont platanistoid (figure 15). In fact, more than half of the platanistoids of the sample have a TBL smaller than 2.3 m, and the others do not exceed 2.9 m in length except *Macrosqualodelphis*. A significant point is that squalodelphinids are the clade of homodont platanistoids displaying the widest range in TBL, varying from 2.0 m in *Huaridelphis* to 3.5 m in *Macrosqualodelphis*, with at least two evolutionary shifts to a smaller size and one to a larger size. This wide range in size is most likely related to the greater diversity of squalodelphinids included in the sample. High diversity in an evolving clade also generates an increase in the maximum body size [67].

Contrasted sizes, combined with other cranial and dental features, are examined below for *Macrosqualodelphis* and the other squalodelphinids from the Chilcatay Fm, in order to understand the ecological significance of the diversity of this fossil platanistoid clade.

### 7.2. Ecological segregation for squalodelphinids of the Chilcatay Fm

**Figure 16.**
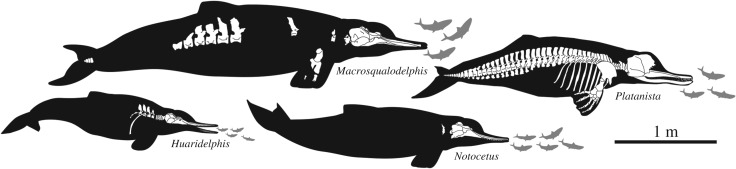
Skeletal remains and inferred body outline of the squalodelphinids from the early Burdigalian of the Chilcatay Fm (Pisco Basin, Peru) and skeletal and body outline of the extant *P. gangetica.* Body lengths based on the Pyenson & Sponberg [30] equation for the fossils and on Jefferson *et al.* [39] for the extant *P. gangetica*.

**Figure 17.**
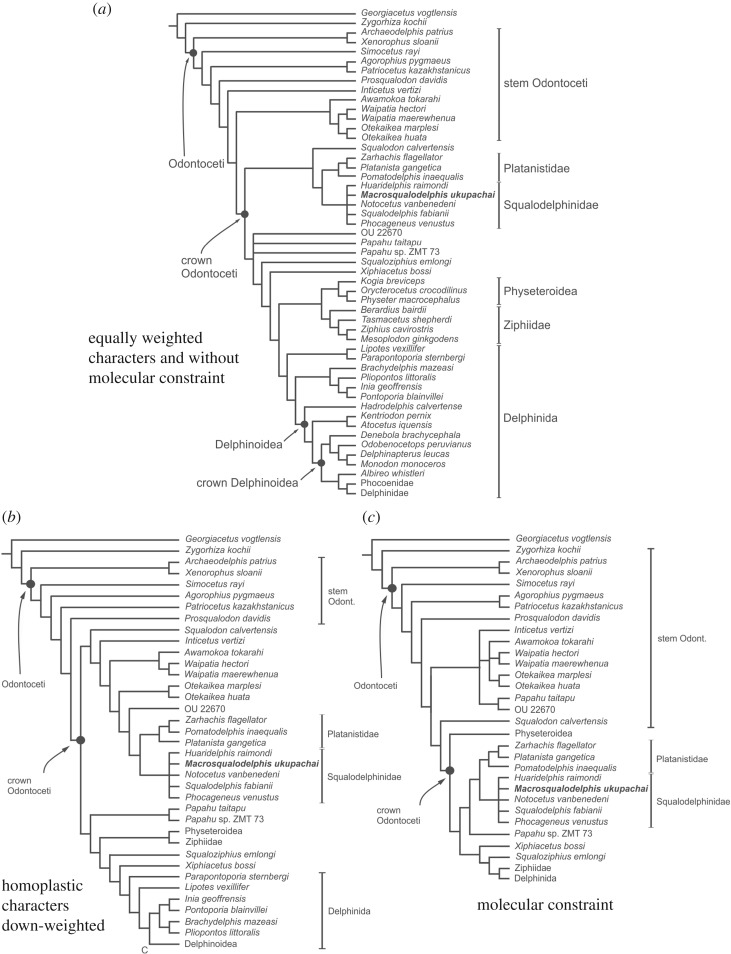
Alternative phylogenetic analyses using a modified matrix of Tanaka & Fordyce [6] and Lambert *et al*. [20]. (*a*) Strict consensus tree for the analysis with equally weighted characters and without molecular constraint; (*b*) strict consensus tree for the analysis with down-weighted homoplastic characters and without molecular constraint; (*c*) strict consensus tree for the analysis with equally weighted homoplastic characters and with molecular constraint (same result with down-weighted homoplastic characters). Note that in all of these analyses: (1) *Macrosqualodelphis* is inside Squalodelphinidae; (2) Squalodelphinidae are a monophyletic group and (3) Platanistidae and Squalodelphinidae are sister groups. The relationships within some clades (e.g. Delphinoidea in tree (*b*) and Delphinidae in tree (*c*)) were not shown because they are not relevant to the position of *Macrosqualodelphis*.

The description of a third squalodelphinid species from the same lithological unit (Chilcatay Fm) and the same geographical region (Pisco Basin) raises the question of how these three related species shared food resources along the western coast of South America during the early Burdigalian. Constituting a key parameter for local diversification, ecological niche segregation among closely related, sympatric species has been investigated in a number of extant cetaceans, including Delphinidae (true dolphins) and Ziphiidae (beaked whales) (e.g. [68–70]). These studies demonstrated that resource partitioning may result from several ecological traits, or combinations of these traits: different foraging habitat (depth, distance to the coast), different behaviour (for example, diel variations in foraging activities) and different feeding ecology (different prey types/position along the local trophic chains). Considering that specimens of *M. ukupachai* were, up to now, not found in the localities of *H. raimondii* and *N. vanbenedeni* (Ullujaya; [13,21], it is tempting to hypothesize a different foraging habitat) (figure 16). In other respects, because they were not discovered in the same locality, there is no evidence that *M. ukupachai* on the one hand and *H. raimondii* and *N. vanbenedeni* on the other hand proceed from exactly the same level in the Chilcatay Fm, and therefore were coeval. If *M. ukupachai* and *H. raimondii* + *N. vanbenedeni* were not contemporaneous, the two groups may very well have had similar foraging habitats without competing. Therefore, our limited sample size, the lack of comparative data about the palaeoenvironmental conditions in various localities of the Chilcatay Fm and the need for an even more refined chronostratigraphic framework should urge for caution when dealing with such considerations. Obviously, diel variations in foraging activities cannot be tested for extinct taxa. Based on comparative skull morphology and dimensions, we thus only assess potential differences in feeding ecology among these three squalodelphinid species. Marked anatomical differences are noted at different levels:

1. *General size*. The BZW of the smallest species, *H. raimondii*, is approximately 80% of the width in the intermediate, *N. vanbenedeni*, and 56% of the width in the largest, *M. ukupachai*, corresponding to highly contrasted estimates for the TBL (2.0, 2.5 and 3.5 m, respectively).
2. *Rostrum shape*. Although not optimally preserved, the rostrum of the holotype of *M. ukupachai* is less tapered, more robust in dorsal view than in the other two species, with the smaller *H. raimondii* displaying the most slender snout.
3. *Size of teeth and tooth count*. Teeth of *M. ukupachai* are proportionally more robust than in the two smaller species, with a greater ratio between maximum tooth diameter and bizygomatic width. Although the tooth count of *M. ukupachai* is unknown, we roughly estimated 15 teeth per row, a value lower than in *N. vanbenedeni* (21–23 teeth per row) and even less than in *H. raimondii* (28–30 teeth per row).
4. *Size of temporal fossa and height of cranial crests*. The temporal fossa is proportionally more voluminous in *M. ukupachai* than in *H. raimondii* and *N. vanbenedeni*, being dorsoventrally higher, anteroposteriorly longer and transversely wider. In addition, the temporal and nuchal crests are more developed in *M. ukupachai* than in *H. raimondii* and *N. vanbenedeni*.

Extending the comparison to the whole family Squalodelphinidae, similar differences are observed, both considering the body size range (see above) and the disparity in cranial and dental morphology (even if the only other squalodelphinid whose rostrum is known is *Squalodelphis*, having the rostrum significantly tapered and 15 teeth per row). Therefore, at a wider geographical scale, the observed squalodelphinid morphological and ecological diversity further illustrates the broad diversification of homodont platanistoids during the Early Miocene crown toothed whale radiation.

Altogether, these anatomical differences between squalodelphinids are reminiscent of differences between several morphotypes of extant delphinids, for example the smaller common dolphin *Delphinus delphis* Linnaeus, 1758 [71] with a more tapered rostrum, higher tooth count, and smaller teeth, the intermediate bottlenose dolphin *Tursiops truncatus* (Montagu, 1821) [72], and the larger false killer whale *Pseudorca crassidens* Owen, 1846 [73] with a much less tapered rostrum, lower tooth count, more robust dentition, larger temporal fossa and higher cranial crests [74–76]. The significantly greater body size of *M. ukupachai* combined with more powerful bites revealed by its cranial and dental features (e.g. [77]) suggest that it was capable of preying upon larger prey items, positioned higher along the local trophic chains, as observed for *P. crassidens* [78,79]. Such an ability to broaden the range of prey sizes constitutes a key parameter for ecological segregation among sympatric modern odontocetes [68–70]; it could be tested in fossil species via stable isotope analyses (e.g. [80,81]).

### 7.3. Squalodelphinid extinction and platanistoid-delphinidan replacement

Whereas squalodelphinids are thought to get extinct before the Middle Miocene, early delphinidans sharing morphological similarities with *M. ukupachai* appear in the early Middle Miocene record; both from Langhian deposits, *Hadrodelphis calvertense* Kellogg, 1955 [82] and *Liolithax pappus* (Kellogg, 1955) [83] are large homodont dolphins displaying a low tooth count, robust teeth, a large temporal fossa and high cranial crests [82–85], suggesting a feeding ecology relatively similar to *M. ukupachai*. Other large generalist predators from the delphinidan clade are known from the Serravallian and Pliocene (e.g. *Macrokentriodon morani* Dawson, 1996 [84], the delphinids *Hemisyntrachelus cortesii* (Fischer, 1829) [86] and *Orcinus citoniensis* Capellini, 1883 [84,87–89]), further supporting the scenario of a replacement of platanistoids (including squalodelphinids) by delphinidans in various marine ecological niches during the Miocene [18]. Interestingly, an apparent Late Miocene gap in the record of large delphinidan generalist predators could have been partly filled by macroraptorial sperm whales (e.g. [90,91]).

## 8. Conclusion

*Macrosqualodelphis ukupachai* is a new species of the extinct platanistoid family Squalodelphinidae based on a well-preserved partial skeleton collected from the Early Miocene (*ca* 19–18 Ma) fossiliferous beds of the Chilcatay Fm outcropping in the Western Ica Valley (southern coast of Peru). The age of this skeleton is further constrained via ^40^Ar/^39^Ar dating of a local volcanic ash layer to 18.78 ± 0.08 Ma (early Burdigalian).

Our phylogenetic analysis supports the referral of *M. ukupachai* to the monophyletic family Squalodelphinidae, of which it constitutes the earliest diverging lineage.

The main distinctive character of *M. ukupachai* is its large size: its estimated TBL is approximately 3.5 m, significantly larger than all other known squalodelphinids, including *N. vanbenedeni* (2.5 m) and *H. raimondii* (2.0 m), both also found in the Chilcatay Fm. Combined with cranial and dental features (robust rostrum less tapered than in other squalodelphinids, large temporal fossa, prominent nuchal and temporal crests, and more robust teeth), the large body size of *M. ukupachai* suggests that this squalodelphinid was able to prey upon larger prey items. Consequently, *M. ukupachai* would have been positioned higher along the local trophic chain than the roughly contemporaneous *N. vanbenedeni* and *H. raimondii*. Therefore, it is suggested that the squalodelphinid diversity, both locally and worldwide, could be related to their partition into different dietary niches, as is observed in the extant delphinids.

This new record further illustrates the first, Early Miocene, broad radiation of crown odontocetes in marine environments, with a major contribution of homodont platanistoids. This Early Miocene morphological and ecological diversification of platanistoids (including squalodelphinids) was followed by the radiation of delphinidans (porpoises, true dolphins and relatives) during the Middle–Late Miocene. The only extant survivor of the platanistoid ‘golden age’ is the endangered South Asian river dolphin *P. gangetica*, confined in freshwater ecosystems of the Ganges, Indus and Brahmaputra river basins.

## Supplementary Material

Nexus file for the main phylogenetic analysis

## Supplementary Material

Nexus file for an alternative phylogenetic analysis (1)

## Supplementary Material

Nexus file for an alternative phylogenetic analysis (2)

## Figures and Tables

**Figure 18.**
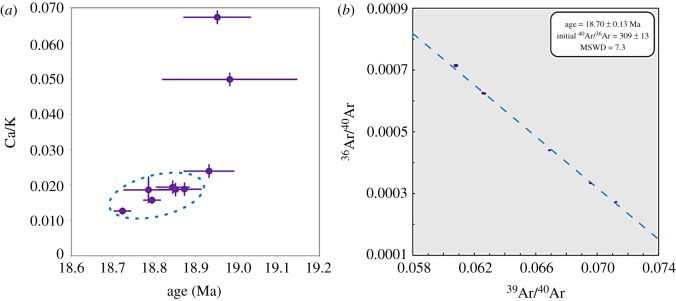
Ca/K versus age correlation diagram and isochron. (*a*) Ca/K versus age correlation diagram of the CHILC-AT1 biotite separate obtained by ^40^Ar/^39^Ar analyses. The dashed ellipse includes the isochemical heating steps 4–9 considered for the age calculations. Step 9 is the one with the lowest Ca/K ratio and the youngest age. Other steps with a high Ca/K ratio have been considered as a result of alteration and not taken into account in the age calculation. All uncertainties are given as 2*σ*. (*b*) Isochron of the CHILC-AT1 biotite crystals, considering only the isochemical heating steps 4–9. The age obtained is 18.70 ± 0.13 Ma, with an MSWD of 7.3. The sizes of the ellipses representing points are the 2*σ* uncertainties.

## Data accessibility

This published work and the nomenclatural acts it contains have been registered in ZooBank. The LSID for this publication is: zoobank.org:pub:939E6EEF-6DC7-4AEF-B033-8A510535FE12. All other relevant supporting data are reported in the appendices below and in the electronic supplementary material.

## Appendix A. Characters and matrix used for the phylogenetic analysis

List of characters for the phylogenetic analysis

Characters are polarized with respect to *Zygorhiza* as the outgroup (table 3). Adapted from [13,15].

1. Rostrum elongation ([92], modified): short, ratio between rostrum length and CBL <0.70 (0); elongated, ratio >0.70 (1).
2. Apex of the rostrum constituted only by the premaxillae on more than 10% of its total length and lacking alveoli [93]: absent (0), present (1).
3. Lateral rostral suture between premaxilla and maxilla deeply grooved [41]: no (0); yes (1).
4. Marked asymmetry of the premaxillae on the rostrum, at some distance anterior to the premaxillary foramina, with the right premaxilla distinctly narrower than the left in dorsal view: absent (0); present (1).
5. Widening of the premaxillae at the rostrum base: narrow premaxillae, ratio between the width of the rostrum and the transverse width of the premaxillae at the antorbital notch <0.60 (0); wide premaxillae, ratio between 0.60 and 0.75 (1); extremely wide premaxillae nearly reaching the lateral margin of the rostrum, ratio >0.75 (2).
6. Dorsal opening of the mesorostral groove anterior to the rostrum base ([94], modified): narrower than the premaxilla (0); wider than the premaxilla (1).
7. Deep, V-shaped, left antorbital notch, related to an anteriorly pointed antorbital process: no (0); yes (1).
8. Elevated antorbital region, distinctly higher than the dorsal margin of the rostrum base in lateral view: no (0); yes (1).
9. Distinct dorsal crest in the antorbital–supraorbital region: no (0); yes (1).
10. Thickening of the antorbital process of the frontal, quantified as a ratio between the height of this process measured in lateral view perpendicular to the maxilla-frontal suture and the vertical distance from the lower margin of the occipital condyles to the vertex of the skull; absent, ratio <0.25 (0); present, ratio >0.30 (1).
11. Widening of the cranium: cranium roughly as long as wide or longer than wide with ratio between cranium length (longitudinal, from occipital condyles to the level of antorbital notches) and postorbital width >0.90 (0); cranium distinctly shorter than wide with ratio <0.90 (1).
12. Posterior infraorbital foramen(ina) along the vertex more medial than the lateralmost margin of the premaxilla in the cranium: no (0); yes (1).
13. Deep fossa in the frontal on orbit roof, at the level of the frontal groove: no (0); yes (1).
14. Vertex distinctly shifted to the left compared to the sagittal plane of the skull: no (0); yes (1).
15. Transverse premaxillary crest on the vertex [93]: absent (0), present (1).
16. Ventral exposure of the palatine ([95], modified): palatine widely exposed anterior to the pterygoid (0); palatine only exposed laterally to the lateral lamina of the pterygoid (1); palatine completely covered by the pterygoid (2).
17. Hamular fossa of the pterygoid sinus [13]: short, not reaching anteriorly the level of the antorbital notch (0); long, extending anteriorly on the palatal surface of the rostrum (1).
18. Thickening of the zygomatic process of the squamosal; absent, ratio between the maximum distance from the anteroventral margin of the zygomatic process to the posterodorsal margin, in lateral view, and the vertical distance from the lower margin of the occipital condyles to the vertex of the skull <0.35 (0); present, ratio >0.35 (1).
19. Circle-shaped dorsal outline of the zygomatic process of the squamosal in lateral view: no (0); yes (1).
20. Articular rim on the lateral surface of the periotic ([95], modified): absent (0); present (1); present and hook-like (2).
21. Pars cochlearis of the periotic square-shaped in ventral view [95]: no (0); yes (1).
22. Aperture of the cochlear aqueduct of the periotic ([95], modified, [96]): small (0); very small (1); large and thin-edged (2).
23. Aperture of the cochlear aqueduct of the periotic ([95], modified): faces mediodorsally (0); faces dorsally (1).
24. Transverse thickening of the anterior process of the periotic [95]: no (0); yes (1).)
25. Internal auditory meatus of the periotic oval, with the dorsal opening for the facial canal lateral to the spiral cribriform tract [97]: no (0); yes (1).
26. Separate ossicle at the apex of the anterior process of the periotic [97]: no (0); yes (1).
27. Elongated anterior spine on the tympanic bulla, associated with a marked anterolateral convexity [95]: no (0); yes (1).
28. Ventral groove of the tympanic affecting the whole length of the bone, including the anterior spine [95]: no (0); yes (1).
29. Extent of the inner and outer posterior prominences of the tympanic: both prominences with approximately the same posterior extent (0); outer posterior prominence posteriorly longer than the inner posterior prominence (1); outer posterior prominence posteriorly shorter than the inner posterior prominence (2).
30. Dorsal margin of the involucrum of the tympanic cut by a median indentation, in medial view [46]: absent (0), present (1).
31. Apical extension of the manubrium of the malleus [95]: no (0); yes (1).
32. Loss of double-rooted posterior teeth: [95]: no (0); yes (1).
33. Retention of accessory denticles on posterior teeth [96, modified]: yes (0); no (1).
34. Tooth count per upper or lower row: <25 (0); >25 and <33 (1); >33 (2).
35. Strong development of the dorsal transverse process of the atlas and extreme reduction of its ventral process [95]: no (0); yes (1).
36. Great reduction of coracoid process of the scapula [95,96,98]: no (0); yes (1).
37. Great reduction or loss of supraspinatus fossa, with acromion located on anterior edge of scapula [95,96,98]: no (0); yes (1).
38. Deep lateral groove on mandible [99]: no (0); yes (1).
39. Medial margin of the antorbital notch made of a thin plate: (0) no, robust lateral margin of the rostrum at base; yes (1).
40. Dorsal surface of vertex: flat (0); markedly transversely and longitudinally convex (1).
41. Vertex strongly transversely pinched: absent (0); present, maxillae converging markedly posterior to bony nares (1).
42. Left frontal longitudinally shorter than the right frontal at the vertex, due to the shift of the vertex on the right side: absent (0); present (1).

**Table 3.** Data matrix of 42 characters for one outgroup (*Zygorhiza*), 17 platanistoids with single-rooted posterior teeth (Allodelphinidae, Squalodelphinidae and Platanistidae), and other possibly related odontocetes (*Squalodon*, *Waipatia* and the eurhinodelphinids *Eurhinodelphis*, *Xiphiacetus* and *Ziphiodelphis*). All characters are treated as unordered; 0, primitive state; 1, 2, derived states; a, variable between 0 and 1; ?, missing character. Adapted from [13] and [15].

	5	10	15	20	25	30	35	40	
*Zygorhiza*	00000	00000	00000	00000	00000	00000	00000	000?0	?0
*Squalodon*	10000	00000	00000	00000	00000	00000	00000	11000	00
*Waipatia*	00001	00000	00000	00000	00000	00000	00000	??000	01
*Xiphiacetus*	11101	001a0	10000	000a0	00000	00011	?1020	00100	01
*Eurhinodelphis*	11101	00000	10001	00000	00000	00011	01?20	???00	01
*Ziphiodelphis*	11101	00100	10001	00000	00000	00011	01020	??100	01
*Zarhinocetus*	10100	10100	10010	0100?	00000	0???0	?1?2?	??100	10
*Allodelphis*	10100	10000	10000	01001	10000	01020	?1?20	??a?0	10
*Ninjadelphis*	10??0	a1000	10000	0100?	10000	01020	?1?20	10100	1?
*Macrosqualodelphis*	00011	00100	1?110	1111?	?????	?????	?1?01	???00	00
MUSM 603	??011	01100	1??10	?1111	1??0?	01020	01???	??00?	??
*Huaridelphis*	00011	01100	11110	11111	12100	0???0	?1011	???00	01
*Notocetus*	00011	01100	11110	11111	12100	01100	11001	11?00	01
*Squalodelphis*	00012	11100	1?110	?1101	12100	01100	1100?	??0??	01
*Phocageneus*	?????	?????	?????	????1	12100	01100	110?1	?????	??
*Medocinia*	??012	11101	1?110	1110?	?????	?????	?1???	???00	0?
USNM 475496	??0??	?1101	11110	?110?	?????	?????	?10??	?????	0?
*Araeodelphis*	00001	0010?	?1110	???0?	?????	?????	?102?	??111	01
*Dilophodelphis*	00101	01111	11110	?1111	0??10	0????	?1?2?	???1?	10
*Zarhachis*	10111	00111	11110	11102	00010	01010	01120	??111	01
*Pomatodelphis*	10111	00111	11110	11102	00010	01010	01120	??1?0	00
MUSM 1611	?????	?????	?????	????2	01001	1????	?????	?????	??
*Platanista*	00111	00010	11110	21102	01001	11010	01110	11111	11

## Appendix B. Alternative phylogenetic analyses using a modified matrix of Tanaka & Fordyce [6] and Lambert *et al*. [20] (table 4 and figure 17)

**Table 4.** Coding of characters for *Huaridelphis*, *Macrosqualodelphis*, *Notocetus* and *Squalodelphis* in the matrix of Tanaka & Fordyce [6], modified by Lambert *et al*. [20]. *Huaridelphis* and *Macrosqualodelphis* were not considered in these previous analyses, whereas *Notocetus* and *Squalodelphis* are here coded differently for some characters. 0, primitive state; 1–6 derived states; a, variable between 0 and 1; b, between 1 and 2; c, between 0 and 2; and d, between 2 and 3; ?, missing character; -, character inapplicable.

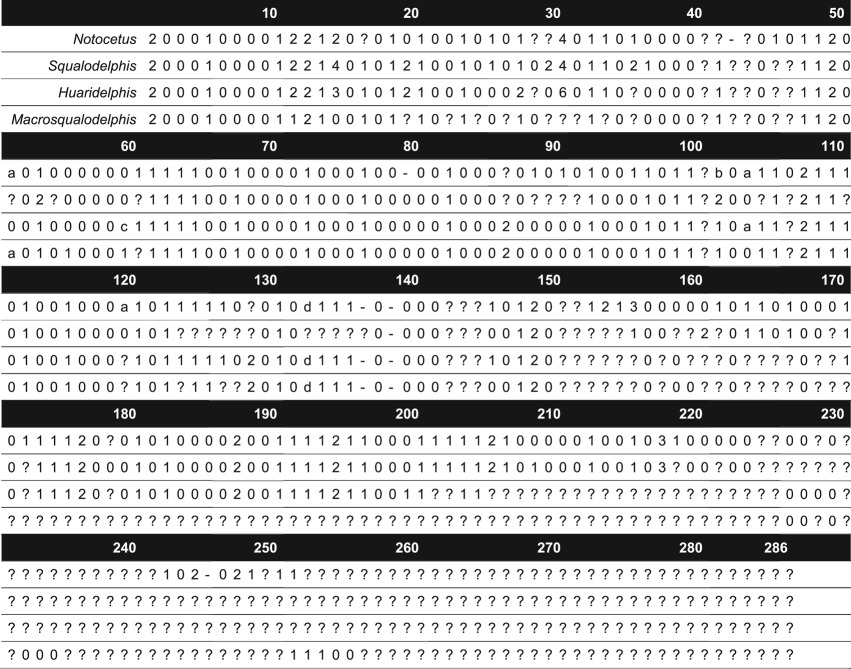

## Appendix C. ^40^Ar/^39^Ar results of the tephra layer

**Table 5.** ^40^Ar/^39^Ar step-heating results of the CHILC-AT1 biotite separate. All isotopes are in ml; ages are in Ma. All uncertainties are given as 1 s.d.

step	*T* (°C)	^40^Ar total	error ^40^Ar	^40^Ar*	^39^Ar	error ^39^Ar	% ^39^Ar	^38^Ar	error ^38^Ar	^38^ArCl	^37^Ar	error ^37^Ar
1	712	3.44 × 10^−8^	1.35 × 10^−11^	1.17 × 10^−9^	1.46 × 10^−10^	3.70 × 10^−13^	1.91	5.94 × 10^−11^	6.71 × 10^−14^	3.67 × 10^−11^	2.01 × 10^−11^	2.29 × 10^−13^
2	818	1.61 × 10^−8^	4.94 × 10^−12^	5.32 × 10^−9^	4.07 × 10^−10^	3.64 × 10^−13^	5.32	6.37 × 10^−11^	6.74 × 10^−14^	5.21 × 10^−11^	1.04 × 10^−11^	2.25 × 10^−13^
3	903	6.83 × 10^−9^	1.27 × 10^−12^	5.12 × 10^−9^	3.92 × 10^−10^	4.07 × 10^−13^	5.14	5.05 × 10^−11^	5.51 × 10^−14^	4.48 × 10^−11^	4.84 × 10^−12^	2.24 × 10^−13^
4	942	4.60 × 10^−9^	9.62 × 10^−13^	3.62 × 10^−9^	2.79 × 10^−10^	3.86 × 10^−13^	3.66	3.44 × 10^−11^	4.08 × 10^−14^	3.05 × 10^−11^	2.68 × 10^−12^	2.29 × 10^−13^
5	995	8.73 × 10^−9^	2.31 × 10^−12^	7.10 × 10^−9^	5.46 × 10^−10^	4.87 × 10^−13^	7.15	6.74 × 10^−11^	7.05 × 10^−14^	6.00 × 10^−11^	5.30 × 10^−12^	2.29 × 10^−13^
6	1035	1.15 × 10^−8^	1.46 × 10^−12^	9.39 × 10^−9^	7.23 × 10^−10^	4.95 × 10^−13^	9.46	8.83 × 10^−11^	9.31 × 10^−14^	7.84 × 10^−11^	7.22 × 10^−12^	2.30 × 10^−13^
7	1076	1.01 × 10^−8^	1.38 × 10^−12^	8.81 × 10^−9^	6.79 × 10^−10^	4.81 × 10^−13^	8.88	8.28 × 10^−11^	8.86 × 10^−14^	7.39 × 10^−11^	6.55 × 10^−12^	2.29 × 10^−13^
8	1117	1.63 × 10^−8^	3.12 × 10^−12^	1.47 × 10^−8^	1.13 × 10^−9^	6.86 × 10^−13^	14.82	1.38 × 10^−10^	1.45 × 10^−13^	1.24 × 10^−10^	9.17 × 10^−12^	2.31 × 10^−13^
9	1163	3.49 × 10^−8^	9.88 × 10^−12^	3.20 × 10^−8^	2.48 × 10^−9^	1.47 × 10^−12^	32.48	3.02 × 10^−10^	3.09 × 10^−13^	2.71 × 10^−10^	1.62 × 10^−11^	2.33 × 10^−13^
10	1289	2.29 × 10^−8^	3.90 × 10^−12^	1.12 × 10^−8^	8.55 × 10^−10^	5.74 × 10^−13^	11.19	1.14 × 10^−10^	1.14 × 10^−13^	9.65 × 10^−11^	2.97 × 10^−11^	2.37 × 10^−13^
step	^36^Ar	error ^36^Ar	age	error age	Ca/K	error Ca/K	Cl/K	error Cl/K	^39^Ar/^40^Ar	sig. 9	^36^Ar/^40^Ar	sig. 6
1	1.11 × 10^−10^	2.14 × 10^−13^	11.70	0.65	0.2682	0.0031	0.0448	0.00013	4.24 × 10^−3^	1.09 × 10^−5^	3.24 × 10^−3^	6.35 × 10^−6^
2	3.62 × 10^−11^	7.16 × 10^−14^	18.98	0.08	0.0498	0.0011	0.0228	0.00002	2.52 × 10^−2^	2.39 × 10^−5^	2.24 × 10^−3^	4.50 × 10^−6^
3	5.75 × 10^−12^	1.53 × 10^−14^	18.93	0.03	0.0239	0.0011	0.0203	0.00002	5.74 × 10^−2^	6.05 × 10^−5^	8.41 × 10^−4^	2.24 × 10^−6^
4	3.28 × 10^−12^	1.15 × 10^−14^	18.79	0.03	0.0186	0.0016	0.0194	0.00003	6.08 × 10^−2^	8.49 × 10^−5^	7.14 × 10^−4^	2.50 × 10^−6^
5	5.46 × 10^−12^	1.39 × 10^−14^	18.87	0.02	0.0188	0.0008	0.0195	0.00002	6.26 × 10^−2^	5.81 × 10^−5^	6.25 × 10^−4^	1.60 × 10^−6^
6	7.21 × 10^−12^	1.97 × 10^−14^	18.85	0.02	0.0194	0.0006	0.0193	0.00001	6.27 × 10^−2^	4.36 × 10^−5^	6.24 × 10^−4^	1.71 × 10^−6^
7	4.47 × 10^−12^	1.44 × 10^−14^	18.85	0.02	0.0187	0.0007	0.0194	0.00002	6.69 × 10^−2^	4.83 × 10^−5^	4.41 × 10^−4^	1.42 × 10^−6^
8	5.43 × 10^−12^	1.53 × 10^−14^	18.80	0.01	0.0157	0.0004	0.0195	0.00001	6.96 × 10^−2^	4.42 × 10^−5^	3.34 × 10^−4^	9.41 × 10^−7^
9	9.54 × 10^−12^	2.45 × 10^−14^	18.72	0.01	0.0126	0.0002	0.0194	0.00001	7.12 × 10^−2^	4.67 × 10^−5^	2.74 × 10^−4^	7.07 × 10^−7^
10	3.94 × 10^−11^	7.78 × 10^−14^	18.95	0.04	0.0673	0.0005	0.0201	0.00001	3.73 × 10^−2^	2.59 × 10^−5^	1.72 × 10^−3^	3.41 × 10^−6^
